# Local Thermal Adaptation in an Insect‐Transmitted Plant Pathogen: The Role of Virulence Trade‐Offs

**DOI:** 10.1111/eva.70303

**Published:** 2026-07-15

**Authors:** Monica A. Donegan, Josephine Nuño, Ranlin Liu, Leonardo De La Fuente, Rodrigo P. P. Almeida

**Affiliations:** ^1^ Department of Environmental Science, Policy, and Management University of California‐Berkeley Berkeley California USA; ^2^ Department of Entomology and Plant Pathology Auburn University Auburn Alabama USA

**Keywords:** bacterial plant pathogen, countergradient variation, fitness trade‐off, local adaptation, reciprocal transplant, thermal adaptation, virulence‐transmission trade‐off

## Abstract

Little is known about local adaptation to temperature in bacterial pathogen populations in nature. For plant‐pathogenic bacteria, local thermal adaptation may influence the evolution of virulence, which has strong implications for disease management and mitigation. *Xylella fastidiosa* (*Xf*) is an insect‐transmitted bacterial pathogen that infects over 700 plant species globally and causes Pierce's disease of grapevines. Here, we compared in vitro growth and *in planta* virulence of multiple strains from two genetically distinct *Xf* populations from California regions having either a colder climate (Hopland) or warmer climate (Bakersfield). *Xf* strains from a warmer climate grew faster than cold‐climate strains under suboptimal (20°C) temperatures, suggesting a lower thermal optimum. In reciprocal field experiments, infections of plants by *Xf* strains from a colder climate were more likely to survive the winter. After a mild winter in Bakersfield (warmer climate), there were severe symptoms and some vine mortality the following growing season in vines infected with the non‐local, cold‐climate (Hopland) strains, pointing to a potential trade‐off between virulence and transmission due to vine mortality. Our data suggest that *Xf* populations in CA are locally adapted to temperature, with cold‐climate strains exhibiting hypervirulence when transplanted to warmer climates, whereas warm‐climate strains survive at lower rates when transplanted to colder climates. Thus, local thermal adaptation in bacterial pathogens may influence the optimum virulence phenotype across the landscape.

## 1 Introduction

The question of how thermal adaptation varies across microbial populations in space is fundamental to evolutionary ecology. Local adaptation to temperature in microbes –especially bacteria– is understudied (Kraemer and Boynton 2017; Wadgymar et al. 2022). Divergent selection on populations across a temperature gradient can lead to local adaptation, where local ecotypes outperform foreign ecotypes (Kawecki and Ebert 2004), and involve fitness trade‐offs, that is, a fitness cost in non‐local environments. For macroorganisms, there are large scale patterns of increasing cold tolerance and decreasing heat tolerance as latitude increases (Sunday et al. 2019). Yet, such empirical data on microbial thermal adaptation patterns is lacking but is vital to predicting responses to climate change, especially in pathogenic or parasitic species that cause global diseases.

One class of local adaptation is countergradient variation, which also results from divergent selection. In cases of countergradient variation, local genotypes and environments have opposite effects on phenotypes along a gradient (Conover and Schultz 1995). As an example, in the Irish potato famine pathogen, *Phytophthora infestans* , populations from colder climates have a faster growth rate in vitro than populations from warmer climates, even at optimum temperatures (Yang et al. 2016). By contrast, classical local adaptation would predict that populations from warmer climates grow more quickly than populations from colder climates in their optimum warm climate if faster growth bestows a selective advantage. Countergradient variation–like a faster growth rate in colder climates–can be an evolutionary mechanism to compensate for low‐resource conditions or short growing seasons (Conover and Schultz 1995; Fangue et al. 2009; Sefbom et al. 2023). Classical local adaptation and countergradient variation are not mutually exclusive, but in some cases one ecotype can outperform another in both local and non‐local environments. In experimental evolution of *E. coli* , there can be costs to adaptation to high and low temperatures in vitro, but fitness trade‐offs are not universal (Bennett et al. 1992; Bennett and Lenski 2007).

Thermal adaptation patterns are particularly relevant to the spread of plant pathogens in both agricultural and natural systems. The geographic ranges of plant pathogens are shifting under climate change (Singh et al. 2023), and increasing disease pressure may affect global food security and primary productivity. However, local thermal adaptation studies have focused primarily on non‐bacterial taxa, and there are only a few studies (Laine 2008; Mboup et al. 2012) moving beyond the lab to test hypotheses with plants in the field. Instead, growth rate or temperature sensitivity in vitro as well as lab‐based virulence assays have served as fitness proxies across a temperature gradient (Robin et al. 2017; Yang et al. 2016; Zhan and McDonald 2011). In fungal and oomycete pathogens, there are examples of classical local thermal adaptation (Mariette et al. 2016; Mboup et al. 2012; Stefansson et al. 2013; Zhan and McDonald 2011), as well as local adaptation resulting from countergradient variation (Chen et al. 2017; Yang et al. 2016).

Virulence *in planta* is usually positively correlated with plant pathogen fitness (Sacristán and García‐Arenal 2008), but not always. Here, we define virulence as the quantitative effect of a pathogen on its host, and pathogenicity as the qualitative ability of a pathogen to infect its host (Sacristán and García‐Arenal 2008). There is theoretical and empirical support for selection against hypervirulence in pathogens; intermediate virulence can optimize pathogen fitness due to virulence‐transmission trade‐offs (Anderson and May 1972; Ewald 1983; Frank 1996). Killing a host plant too quickly can be detrimental for plant pathogens, particularly in susceptible hosts (Laine and Barrès 2013), because it limits pathogen spread. Virulence‐transmission trade‐offs are also dependent on the mode of transmission. Insect‐transmitted pathogens differ from vertically transmitted pathogens (Ewald 1983; Froissart et al. 2010; Power and Irwin 1992); optimal virulence for an insect‐borne pathogen also depends on vector behavior and seasonality. Many plant pathogens indirectly modify insect behavior so that infected plants become more attractive to vectors (Eigenbrode et al. 2018). While virulence‐transmission trade‐offs are well established, most empirical support has come from experiments in controlled environments (Turner et al. 2021). The role of environmental variation on virulence evolution in plant pathogens is not yet well defined (Turner et al. 2021).

In the insect‐borne bacterial plant pathogen *Xylella fastidiosa* (*Xf*), temperature influences multiple stages of the disease cycle. *Xf* infects over 700 other plant species (Cavalieri et al. 2025) and causes Pierce's disease of grapevines (PD), a lethal disease that impacts viticulture around the world. Warmer temperatures encourage *Xf* growth (Feil and Purcell 2001), and thus, warmer summers worsen disease symptoms (Burbank et al. 2023; Daugherty et al. 2017). Vector behavior in the *Xf*‐grapevine pathosystem differs from other systems in which vectors prefer symptomatic plants (Eigenbrode et al. 2018). In PD epidemiology, disease symptoms negatively impact transmission, as sharpshooter vectors of *Xf* prefer asymptomatic hosts (Daugherty et al. 2011, 2017). Accordingly, *Xf* attenuates virulence by using cell‐to‐cell signaling to downregulate virulence factors at high cell density (Chatterjee et al. 2008); selection for intermediate virulence may extend the window when plants are asymptomatic, thereby optimizing pathogen spread (Sicard et al. 2018). Since warm temperatures worsen symptoms, selection to modulate virulence may be stronger in warm‐climate *Xf* populations, causing virulence to follow a countergradient pattern.

In the winter, cold temperatures can induce PD overwinter recovery, a phenomenon in which infected grapevines are cured from the bacterial infection when exposed to cold winters (Feil et al. 2003; Lieth et al. 2011; Purcell 1980). The recovery process itself is likely influenced by *Xf* growth in the summer, as grapevines with larger bacterial populations are less likely to recover (Feil et al. 2003; Kahn et al. 2023) (Authors, unpublished). Thus, both summer and winter temperatures can influence *Xf* growth, plant colonization, and disease symptoms, which in turn affect insect transmission and pathogen spread.

Here, our goal was to delineate patterns of thermal adaptation in a bacterial plant pathogen and evaluate potential virulence trade‐offs. We hypothesized that (1) cold‐climate *Xf* strains would survive cold winter temperatures in plants at greater rates than warm‐climate strains and (2) countergradient variation in virulence may result in hypervirulence—and potential trade‐offs with transmission—in cold‐climate strains when transplanted to warmer areas. Previous results from a common garden experiment in a colder California viticultural area support that local (cold‐climate) strains have an advantage over foreign strains in winter survival (Authors, unpublished). We evaluated thermal adaptation in multiple strains from two genetically distinct *Xf* clusters (Donegan et al. 2025; Vanhove et al. 2020): (1) a population in Northern California experiencing warm summers and cold winters and (2) a population in South Central Valley of California experiencing hot summers and cool winters. We compared the growth of strains from both clusters in vitro at different temperatures and performed a reciprocal transplant experiment using infected potted grapevines that were exposed to various winter conditions. To isolate the impact of winter climates, infected vines were grown in a common greenhouse before and after transplantation to field sites for the winter months. Understanding plant pathogen thermal adaptation and its interaction with virulence–transmission trade‐offs have profound applications in disease management and risk assessments.

## 2 Materials and Methods

### 2.1 Strain Selection and Description

We selected 10 *Xf* strains that were previously isolated from unique climates and representative of the main genotype from that location (Donegan et al. 2025; Vanhove et al. 2020). In Bakersfield, there is only one *Xf* genotype, whereas in Hopland, there are two main genotypes circulating. We selected the predominant genotype, which is only found in Hopland; the minority genotype in Hopland is also present in nearby counties like Sonoma. Strains from each group are genetically similar to each other but distinct from those in the other group (Donegan et al. 2025). Five *Xf* strains (Je60, Je66, Je77, Je82, and Je115) were isolated from Bakersfield (Vanhove et al. 2020), a warmer climate in the South Central Valley of California, and five *Xf* strains (D01, D03, D04, D06, and D07) were isolated from Hopland, a colder climate in the North Coast region of California (Donegan et al. 2025). Genomes for these strains are publicly available on NCBI; NCBI identifiers are listed in Table S1. We downloaded daily temperature data from the Sanel Valley station in Hopland and the Arvin‐Edison station in Bakersfield from CIMIS from March 2020 through March 2025 and obtained hourly data from all months of 2024 (State of California 2026). We calculated mean temperature (*T*_mean_) from these 5 years and compared minimum, mean, and maximum temperatures to confirm and illustrate climate differences of these two sites (Figure S1). In the summer, Hopland experiences hot days but cool nights, whereas summer in Bakersfield is constantly hot (Figure S1).

### 2.2 In Vitro Phenotypic Assays

For the 10 strains studied (five from Hopland Cluster “HC”, five from Bakersfield Cluster “BC”), we performed multiple phenotypic measurements at both 20°C (suboptimal) and 28°C (optimal for *Xf*) (Feil and Purcell 2001): (1) cumulative growth curves (2) endpoint biofilm abundance and extent of planktonic growth (3) settling rate and (4) twitching motility. These methods were previously described (Merfa et al. 2023) and summarized below. For each dataset at each temperature, we built a linear mixed model with the *lme4* (Bates et al. 2015) package in R that included strain cluster (BC or HC), day (if applicable), and growth medium (if applicable) as fixed effects, and modeled strain identity (if applicable) and experimental replicate as random effects. Details for all models and results are included in Table S2.

1. *Growth curves:* Strains were grown in liquid PD3 (Davis et al. 1981) broth in 96‐well plates and incubated at 20°C or 28°C. OD_600_ was determined daily for 7 days for plates at 28°C and for 10 days for plates at 20°C. Four independent experiments were carried out (Figure S2). There were 24 replicates per strain, with 8 replicates on each of three 96‐well plates.
2. *Endpoint biofilm and planktonic growth:* These endpoint measurements were conducted on the last day of growth (day 10 for 20°C cultures, day 7 for 28°C cultures) for four independent experiments (Figure S3). There were 24 replicates per strain, with eight replicates on each of three 96‐well plates.
3. *Settling rate:* Strains were initially grown at 28°C for 7 days. Then, strains were incubated at 20°C and 28°C in either PD3 or PW (Davis et al. 1981) liquid media. The change in OD_600_ over 2 h was measured in 2 (for 20°C) or 4 (for 28°C) independent experiments (Figure S4). One replicate per strain was measured in each experiment, so strain identity was not used as a random effect for this statistical modeling.
4. *Twitching motility:* Fringe width of colonies on modified PW plates was measured every other day for 10 days at both 20°C and 28°C for all 10 strains. Two independent experiments were performed (Figure S5). For colonies with no fringe, two photos were captured; for colonies with fringe, six photos were taken. Four measurements of fringe width (μm) were performed for each photo in ImageJ.

### 2.3 Reciprocal Transplant Experiments

We performed two independent reciprocal transplant experiments: from June 2023 to August 2024 and from June 2024 to August 2025. Grapevines were grown from disease‐free cuttings acquired from UC Davis Foundational Plant Services (*n* = 220 in 2023, *n* = 200 in 2024) in a greenhouse at UC Berkeley. Prior to inoculation, all 10 strains were grown on solid PD3 plates for 2 weeks. We mechanically inoculated grapevines by pipetting 10 μL of cell suspension onto two internodes on the lower third of the grapevine stem and then perforated with five pricks of an entomological pin. Vines were tested for pathogen presence using qPCR following an established protocol (Sicard et al. 2020) in August and September and monitored for leaf scorch weekly from August through September. Inoculated vines that tested negative for *Xf* were discarded.

In late September or early October, we transported subsets of infected plants to either Bakersfield or Hopland for the winter (~6.5 months). Plants were kept in screen enclosures at Bakersfield (University of California Cooperative Extension Office, Kern County) or Hopland (Hopland Research and Extension Center). Temperatures were logged hourly in each location during the winter using a Hobo Data Logger. For each year, we calculated cumulative freezing hours (< 0°C), chill hours (< 7.22°C), and fall warm hours (> 20°C) (fall: before Dec. 31st). Following the winter, we returned plants to the Berkeley greenhouse in April. Upon return, we assessed bud break (1/0) status weekly. We evaluated the presence or absence of scorch (1/0) weekly from mid‐June through July, and at the end of the season (late July), we recorded stunting (1/0), scorch (1/0), and bud break (1/0) after 4 months. In late summer, petioles were sampled for pathogen abundance using qPCR.

#### 2.3.1 Experiment #1 (Exp. 1): 2023–2024

We inoculated 220 *Vitis vinifera* cv. Cabernet Sauvignon plants: 20 plants per treatment with 11 treatments (1 negative control and 10 strains). Because of plant availability and bacterial growth, inoculations with the pathogen strains occurred on three separate days: (1) 6/6/23 with D06, D07, Je60, and Je77, (2) 6/8/23 with D01, D04, Je66, Je115, and negative controls, and (3) 7/21/23 with D03 and Je82. While there was an effect of date on inoculation success (Figure S6), strains from each cluster (HC or BC) were paired on each inoculation date to control for date effects. After removing plants for which pathogen inoculation was unsuccessful, 79 and 75 plants were moved to Hopland and Bakersfield, respectively. Vines were overwintered in Bakersfield from 10/3/23 to 4/15/24 and in Hopland from 9/28/23 to 4/23/24. For qPCR testing, we sampled vines in early July. For vines that tested negative, we did additional sampling and testing in late July to confirm plant recovery from *Xf* infections.

#### 2.3.2 Experiment #2 (Exp. 2): 2024–2025

The second experiment included minor changes due to biological constraints. We used the same strains, except for BC strain Je82, which could not be cultured from a freezer stock. Due to plant availability, we inoculated 100 cv. Cabernet Sauvignon plants and 100 cv. Ciliegiolo plants. In June (6/17/24), we mechanically inoculated 200 plants (10 of each vine cultivar per treatment) with the nine *Xf* strains (4 bc, 5 HC) and one saline buffer (negative control). In addition to weekly scorch presence or absence evaluations, we did one evaluation of symptom severity (9/24/24) based on a 0–5 scale described previously (Deyett et al. 2019). We added this measurement in the second experiment because we noticed symptom severity differences that may not be captured in symptom presence or absence. Pathogen abundance was measured with qPCR in early September; vines for which the pathogen was not initially detected were retested in late September.

We transported 72 vines to Hopland from 9/26/24 to 4/22/25 and 75 vines to Bakersfield from 10/3/24 to 4/14/25. In late July 2025, we evaluated symptom severity using a 0–5 scale (Deyett et al. 2019). Petioles for all vines were sampled for qPCR testing in July. Vines for which the pathogen was not initially detected were retested in early August.

### 2.4 qPCR Methods

DNA from plant tissue was extracted using a DNEasy Plant Mini Kit (Qiagen) from 0.1 g combined from two petiole samples for tissue homogenization. All qPCR testing from samples collected in 2023 and 2024 was run on an Applied Biosystems 7500 Fast Real‐Time PCR System machine with previously described *recF1* primers and thermocycling conditions (Sicard et al. 2020). For the 2025 greenhouse season, plates were processed using a Thermo Fisher Scientific QuantStudio 3 Real‐Time PCR instrument. The CT cutoffs were 37 for the 7500 Real‐Time PCR system and 32 for the QuantStudio 3; standard curve equations (to convert CT values to bacterial population estimates i.e., log (CFU/g)) were determined for each machine.

### 2.5 Reciprocal Transplant Data Analysis

All data cleanup, visualization, and analysis were performed in the R programming language, using the following packages: *tidyr* (Wickham et al. 2019), *dplyr* (Wickham, François, et al. 2025), *tidyverse* (Wickham et al. 2019), *ggplot2* (Wickham 2016), *ggrepel* (Slowikowski 2026), *ggsurvfit* (Sjoberg et al. 2026), *car* (Fox and Weisberg 2019), *survival* (Therneau 2020), *scales* (Wickham, Pedersen, and Seidel 2025), and *survminer* (Kassambara et al. 2026). Statistical results and model details are listed in Table S3. Because we were interested in between‐cluster differences, for all models we chose a mixed‐modeling approach, including strain cluster (HC or BC) as a fixed effect and strain identity as a random effect. Models with data from both experimental replicates included experiment as a random effect. All linear mixed models (LMMs) and generalized linear mixed models (GLMMs) were built with *lme4* (Bates et al. 2015) and *glmmTMB* (Brooks et al. 2017; McGillycuddy et al. 2025) packages in R, respectively.

### 2.6 Pre‐Winter Data Models

(1) We built a GLMM with binomial error, using inoculation success as the response variable. The data included all inoculated plants from both experiments, excluding negative controls. Fixed variables included cluster, variety, and inoculation date. (2) Population estimates (log (CFU/g) of bacterial cells) were derived from end‐of‐season CT values; only positive vines from both experiments were included in the dataset. For vines that were tested more than once, we included the maximum CT value. Using an LMM, log scale populations (response) were modeled as a function of cluster and variety. (3) For the second experiment only, we used severity scores (from 0 to 5) from positive vines only before winter as a response variable in an LMM, with cluster and variety as fixed variables, and strain as the only random variable. (4) For each year, we fit a mixed‐effects Cox proportional hazards model of scorch development (days to first observation of scorch symptom) in the fall using the *coxme* package (Therneau 2024). For Exp. 1, there was a fixed effect of cluster and a random effect of strain. For Exp. 2, we added variety in addition to the fixed effect of strain cluster.

### 2.7 Post‐Winter Data Models

(1) Recovery (1/0) was modeled in a GLMM with binomial error, with cluster, variety, winter location, and maximum bacterial population the previous fall (from CT values) as fixed effects. (2) Because estimates of pathogen abundances in the spring of 2024 and 2025 were performed on different qPCR instruments, we split these LMMs by experiment, with only a random effect of strain. The LMM included cluster and location for Exp. 1, with the addition of variety as a fixed effect for Exp. 2. (3) For both scorch and bud break, mixed‐effects Cox proportional hazards models of symptom development (days to first symptom observation) were constructed similarly to that used for pre‐winter scorch. For Exp. 1, there were fixed effects of cluster and location, and a random effect of strain. For Exp. 2, we added a fixed effect for variety. Only vines with positive qPCR results were included in the model. (4) Then, we evaluated bud break, stunting, and scorch at the end of the post‐winter season. For each of these three response variables, we built a GLMM with binomial error and fixed effects of cluster, variety, and location, using only positive vines. (5) In the second experiment, we modeled end‐of‐season severity scores (from 0 to 5) as a response variable in an LMM, with cluster, variety, and location as fixed variables, and strain as the only random variable. Only vines with positive qPCR results were included in this model.

## 3 Results

### 3.1 Counter‐Gradient Variation in Growth Rates In Vitro

**FIGURE 1:**
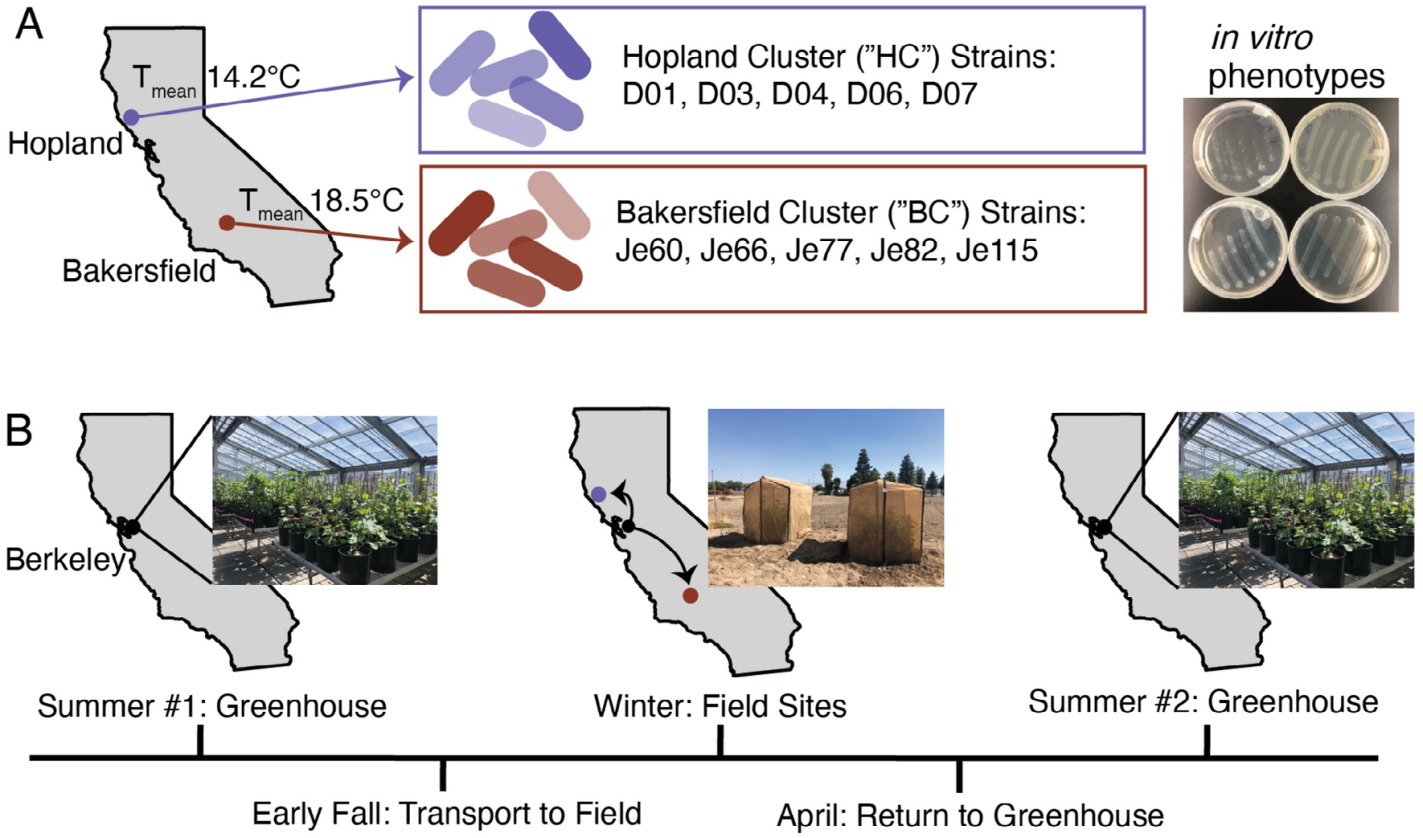
Experimental design (A) We selected five strains from Hopland (“HC”: Hopland Cluster) and five from Bakersfield (“BC”: Hopland Cluster). We measured several in vitro growth phenotypes at 20°C and 28°C. (B) Timeline of reciprocal transplant experiment: We infected vines using the same five strains per cluster as above. The experiment was repeated twice (Exp.1: June 2023–August 2024, Exp. 2: June 2024–August 2025).

**FIGURE 2:**
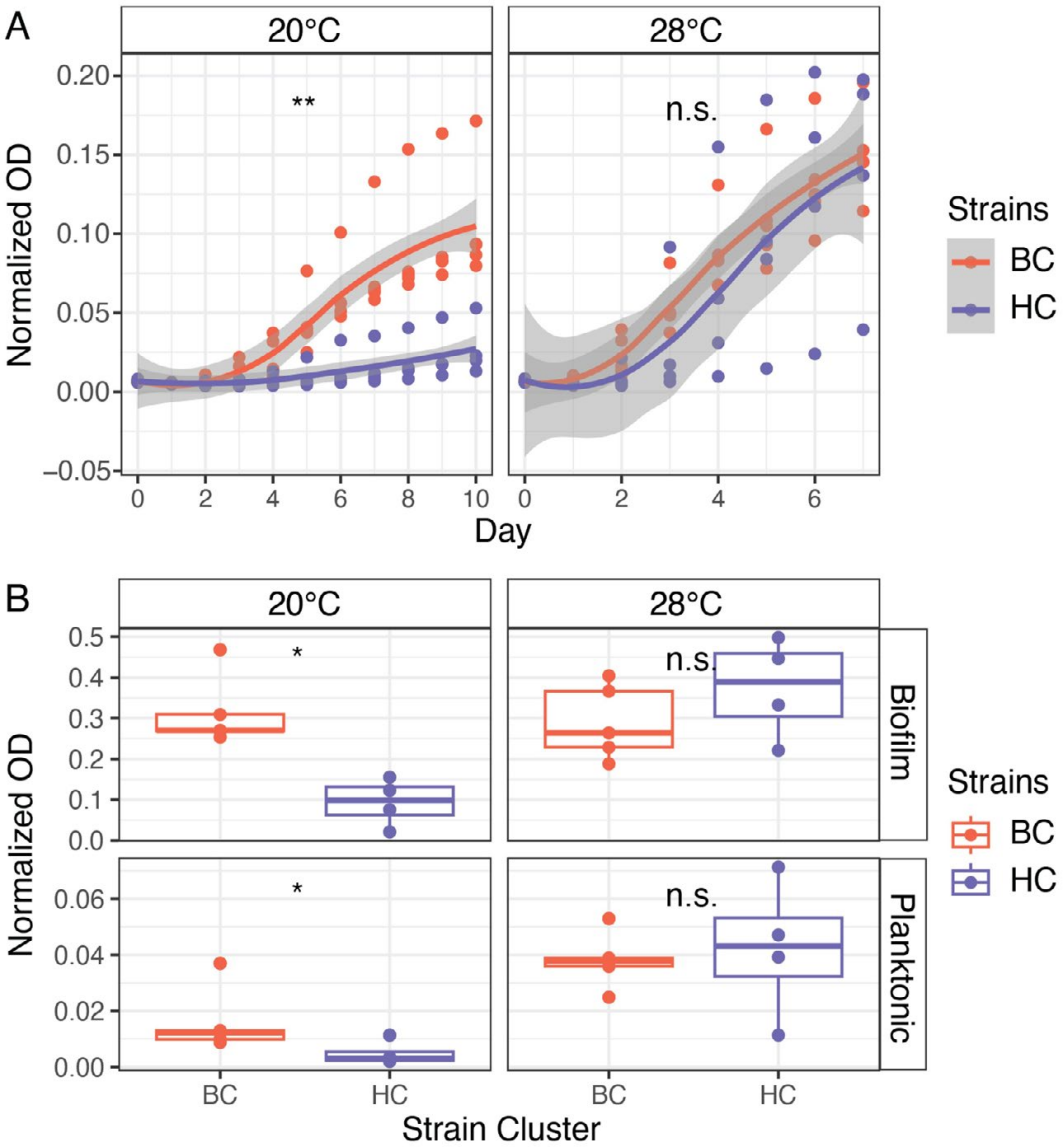
In vitro growth phenotypes. For all experiments, we used five strains per genetic cluster. For each graph, one representative experimental replicate is shown; however, the significance levels are from models using data from all experimental replicates. (A) Hopland Cluster (“HC”) and Bakersfield Cluster (“BC”) strains (one dot per strain) at 20°C and 28°C. (B) Endpoint reading (Day 10) of biofilm formation and planktonic growth at 20°C. Additional data from all experimental replicates are included in Figures S2 and S3. “OD” stands for optimal density.

We chose five *Xf* strains from each genetic cluster (HC: Hopland cluster, BC: Bakersfield cluster) to compare an array of phenotypic traits in vitro at two temperatures (Figure 1A). A temperature of 20°C is generally suboptimal for *Xf* growth, while 28°C is considered optimal (Feil and Purcell 2001). Strains in these clusters originated in locations with climates differing in temperature (Figure 1A, Figure S1); the mean annual temperature (T_mean_) is 14.2°C in Hopland and 18.5°C in Bakersfield. While strains from the two clusters did not differ in growth at 28°C, BC strains showed greater growth at 20°C (𝛸^2^ = 9.397, df = 1, *p* = 0.0022) (Figure 2A, Figure S2). Similarly, neither the abundance of biofilm formation nor the extent of planktonic growth differed between groups at 28°C. BC strains produced more biofilm (*χ* ^2^ = 4.529, df = 1, *p* = 0.033) and planktonic growth (*χ* ^2^ = 5.505, df = 1, *p* = 0.019) at 20°C than the HC strains (Figure 2B, Figure S3). We observed a faster settling rate of HC than BC strains (at 28°C; *χ* ^2^ = 4.990, df = 1, *p* = 0.026). This difference was consistent across two different media and at both temperatures (Figure S4). No between‐cluster differences in twitching motility were observed (Figure S5).

### 3.2 Disease Progression Before Transfer to Winter Conditions

**FIGURE 3:**
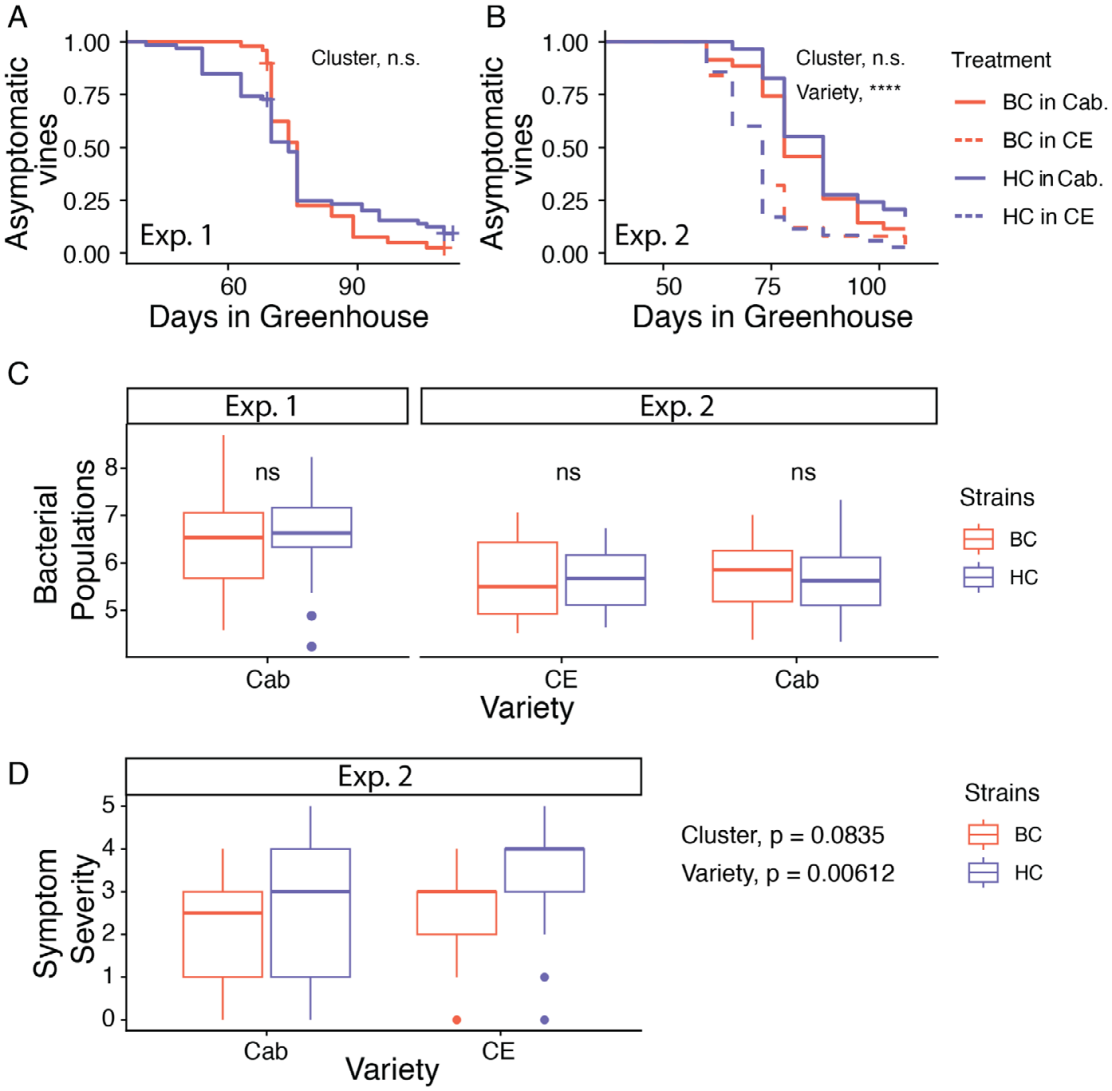
Pre‐winter symptoms and bacterial populations. Pathogen strains are split by genetic cluster: Hopland Cluster (“HC”) and Bakersfield Cluster (“BC”). (A/B) Scorch development over time is graphed as the decline in asymptomatic vines in (A) Exp. 1 and (B) Exp. 2. Only positive plants are included. For Exp. 1, all vines were Cabernet Sauvignon (“Cab.”). In Exp. 2, vines were either Cab. (solid lines) or Ciliegiolo (“CE”, dotted lines). (C) Bacterial populations (log (CFU/g) estimated by qPCR) in positive plants. (D) Symptom severity scored on a 0 to 5 scale (0: no symptoms, 5: most severe) in positive plants in Exp. 2.

In each of two replicate experiments, ca. 200 potted grapevines were inoculated in a greenhouse and incubated for ~3.5 months prior to exposure to different winter conditions in the field (Figure 1B, Figure S8). The incidence of infection did not significantly differ between strain clusters (Figure S6, Table S3). Symptom development rate over time in the greenhouse was similar between the two strain clusters in both experiments (Figure 3, Table S3). In Exp. 2, Ciliegielo vines developed symptoms faster than Cabernet Sauvignon vines (*χ* ^2^ = 24.811, df = 1, *p* < 0.0001, Figure 3B). Bacterial population sizes prior to winter exposure were similar between the two strain clusters and varieties (Figure 3C) (Figure S7). Despite this, in Exp. 2 there were more severe symptoms (0–5 scale) in Ciliegiolo than Cabernet Sauvignon vines (*χ* ^2^ = 7.515, df = 1, *p* = 0.0061, Figure 3D), and slightly more (*p* < 0.10) severe symptoms in HC strains (*χ* ^2^ = 2.995, df = 1, *p* = 0.083) (Figure 3D).

### 3.3 Greater Pathogen Survival of HC Strains

In the fall, all *Xf*‐infected vines were transported to Hopland or Bakersfield and kept outdoors in securely screened‐in, insect‐proof greenhouses from October through April (Figure 1B). Winter temperatures were lower in Hopland than in Bakersfield in both years, assessed by the number of hours of freezing (< 0°C) and chill temperatures (< 7.22°C) (Figure S8). The Exp. 2 winter was colder than that of Exp. 1 in both locations. The fall was warmer (hours > 20°C) in Bakersfield than in Hopland, especially in Exp. 1 (Figure S8).

**FIGURE 4:**
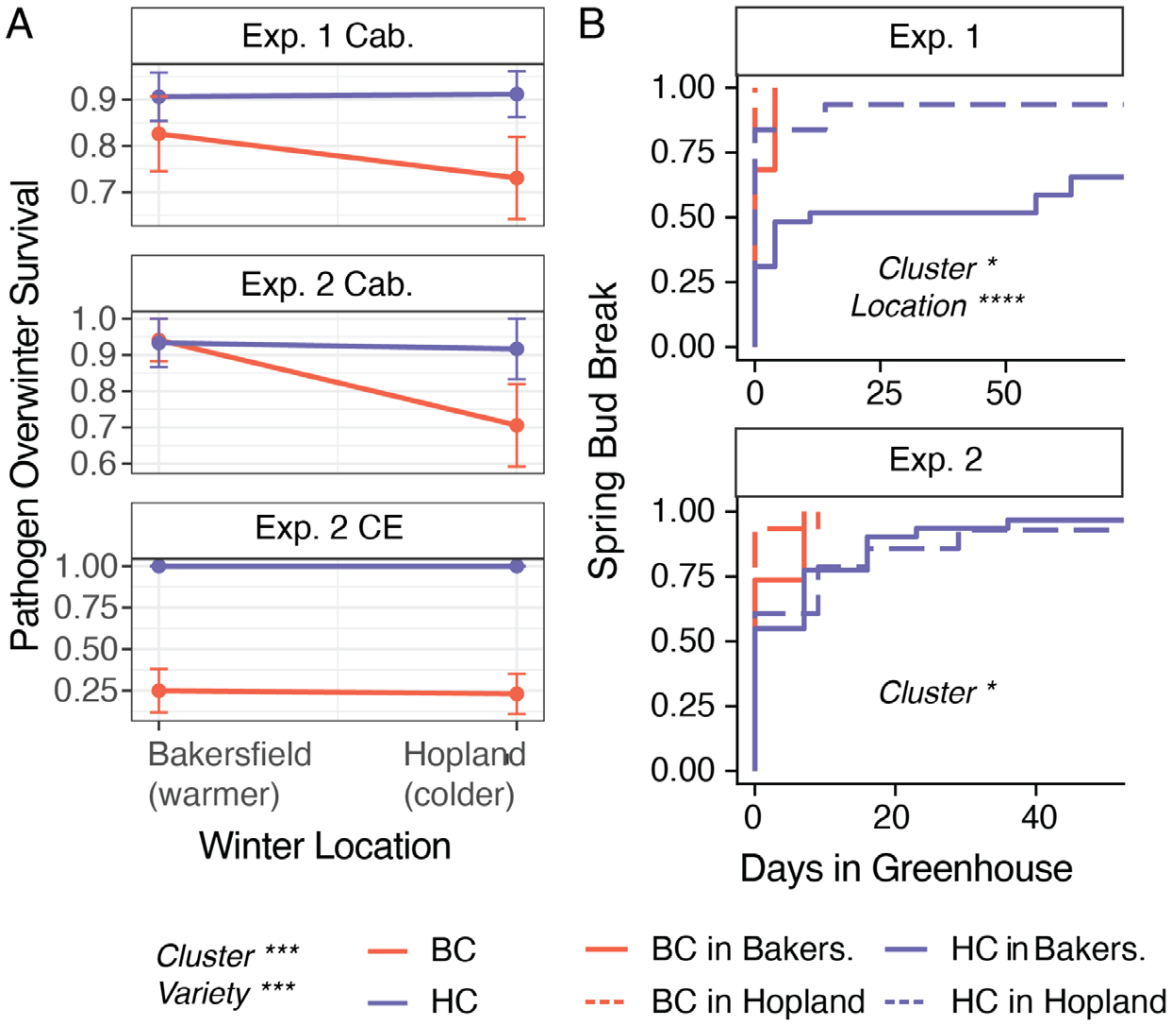
Pathogen overwinter survival and delay in vine bud break after winter treatment Red vs. purple lines show Bakersfield Cluster (“BC”) and Hopland Cluster (“HC”) strains, respectively. (A) Proportion of pathogen survival in vines that were positive the previous fall, split by experiment and variety (“Cab.”: Cabernet Sauvignon, or “CE”: Ciliegiolo). Survival by strain is included in Figure S8. Significant factors (Variety, Cluster) are noted below the graphs. (B) Survival curves of spring bud break in infected vines only (i.e., infections that survived the winter). Line type depicts plants that overwintered in Hopland (dotted) or in Bakersfield (solid). For each experiment, only significant factors are shown with significance levels.

In both years, the incidence of pathogen survival in potted Cabernet Sauvignon vines was generally high (> 70%) (Figure 4A) (Figure S9). There was a slightly greater incidence of survival of HC strains than BC strains in Cabernet Sauvignon vines in Hopland (Figure 4A); that is, the local strains survived at higher rates than the non‐local strains in a colder environment. In Ciliegiolo, almost no BC strain infections survived the winter, while all HC strain infections survived in either location (Figure 4A) (Figure S9). Overall, there was greater pathogen survival of HC strains than BC strains (*χ* ^2^ = 10.910, df = 1, *p* = 0.00096). There were lower rates of pathogen survival in Ciliegiolo vines than in Cabernet vines (*χ* ^2^ = 11.800, df = 1, *p* = 0.00059). Overwintering in a colder location (Hopland) negatively affected pathogen survival (𝛽 = −0.526), but location was not significant (*p* = 0.19).

At the end of the second summer, pathogen populations in infected vines did not differ between strain clusters in Exp. 1 (Figure S10). However, in Exp. 2, there was a significant effect of variety (*χ* ^2^ = 6.349, df = 1, *p* = 0.012) and a near significant effect of cluster (*χ* ^2^ = 3.161, df = 1, *p* = 0.075) on pathogen population size. In Ciliegiolo vines for which the pathogen had survived the winter, the population sizes of BC strains were lower than those of HC strains (Figure S10).

### 3.4 High Incidence of Post‐Winter Symptoms in HC Strain Infections

For all vines, we recorded the occurrence of bud break weekly after plants were returned to the greenhouse from the field in the spring. Bud break was compared only in vines in which the pathogen could still be detected after the winter, since *Xf* infection delays bud break. In Exp. 1, bud break was significantly delayed in vines that overwintered in Bakersfield (*χ* ^2^ = 17.771, df = 1, *p* < 0.0001) and especially in vines infected with HC strains (*χ* ^2^ = 3.966, df = 1, *p* = 0.046) (Figure 4B). In Exp. 2, vines infected with HC strains experienced significantly delayed bud break (*χ* ^2^ = 4.230, df = 1, *p* = 0.040), but neither location nor variety differed significantly in bud break (Figure 4B, Table S3).

**FIGURE 5:**
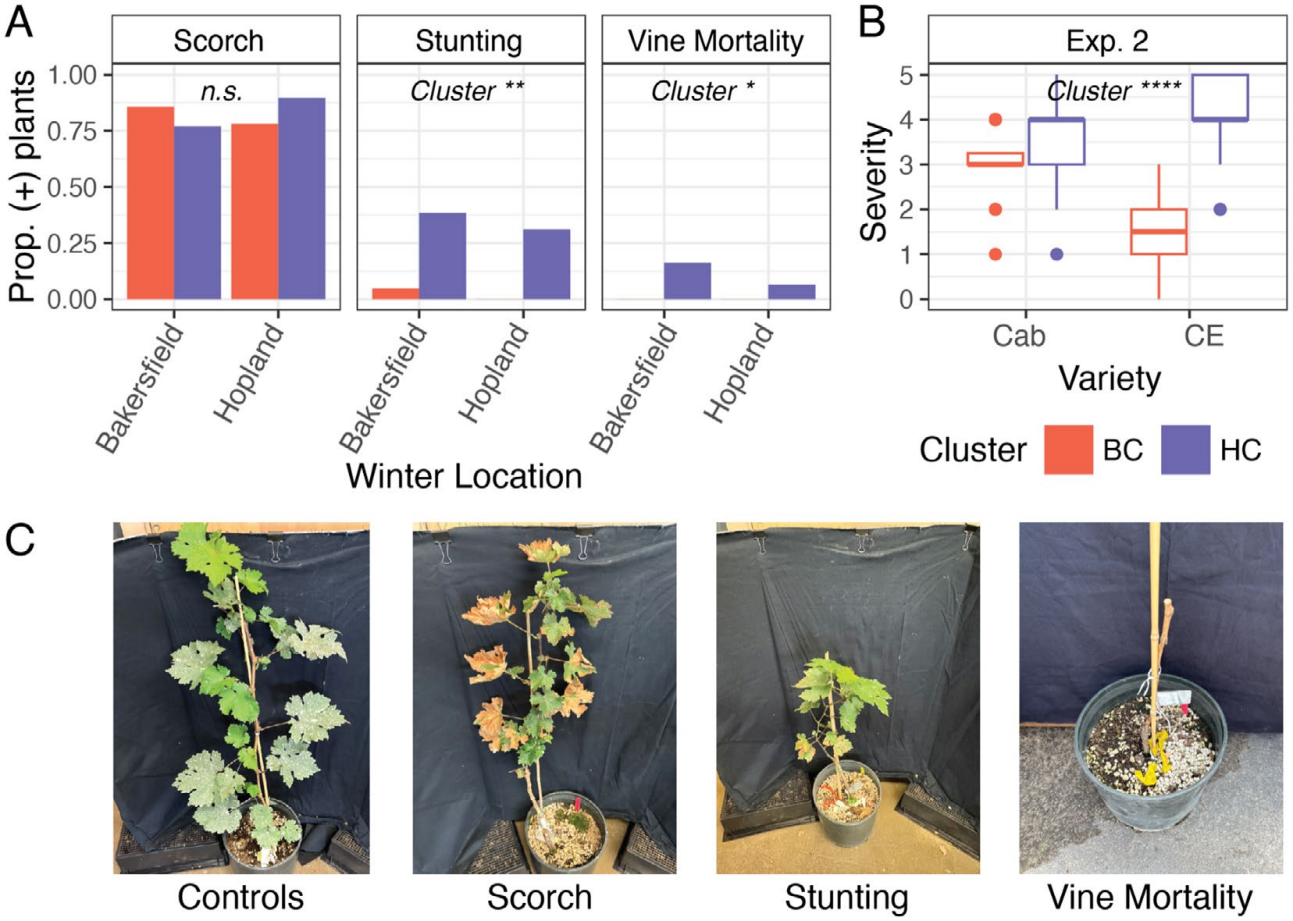
End‐of‐season Symptoms after Winter (A) Proportion of positive plants with each symptom (scorch, stunting, or vine mortality); data is from both experiments. Significance levels are shown for comparison of strain clusters. (B) Symptom severity scored on a 0 to 5 scale (0: no symptoms, 5: most severe) in Exp. 2 at the end of the season. For A and B, the red bars represent Bakersfield Cluster (“BC”) strains, whereas the purple show Hopland Cluster (“HC”) strains. The varieties in the *x*‐axis of B are Cabernet Sauvignon (“Cab”) and Ciliegiolo (“CE”). (C) Photographs of symptom examples in a representative vine.

We evaluated symptoms at the end of each summer following winter exposure (Figure 5). While most infected vines broke buds over the course of the summer, vine mortality was observed in several HC‐infected vines (Figure 5). In Experiment 1, following a very warm fall and mild winter in Bakersfield, most of the dead vines were those that had been infected with HC strains and overwintered in Bakersfield (10 of 31 vines in this strain cluster/location cohort). The incidence of vine mortality was higher in HC‐infected vines (*χ* ^2^ = 6.480, df = 1, *p* = 0.011) and slightly higher in those vines overwintered in Bakersfield compared to Hopland (*χ* ^2^ = 3.311, df = 1, *p* = 0.074).

Additionally, stunted growth was more frequent in vines inoculated with HC strains than BC strains (*χ* ^2^ = 10.980, df = 1, *p* = 0.0009) (Figure 5, Table S3). The end‐of‐season incidence of scorch symptoms (Figure 5) as well as occurrence of scorch development over time (Figure S11) was similar among vines retaining the pathogen following winter exposure, regardless of winter location, variety, or strain cluster (Table S3). While the incidence of scorch symptoms did not differ, symptoms severity (0–5 scale) was higher in HC‐infected vines (*χ* ^2^ = 27.317, df = 1, *p* < 0.001) than in BC‐infected vines in Exp. 2 (Figure 5).

## 4 Discussion

Genetically and geographically separated *Xf* populations showed unique thermal adaptation and virulence phenotypes. We measured greater overwinter pathogen survival and post‐winter symptom incidence and severity in vines infected with cold‐climate than warm‐climate *Xf* strains. These phenotypes are connected through a seasonal cycle: bacterial populations decline during the winter, and the number of surviving bacterial cells in the subsequent spring determines symptom severity. The rate of winter bacterial decline–and therefore symptoms in the subsequent growing season–differed by strain genotype. The conditions for local adaptation were partly met; local *Xf* strains showed better winter survival compared to foreign strains in Hopland, but this pattern was not observed in Bakersfield. Determining the presence of a local vs. foreign advantage in post‐winter symptom severity and incidence hinges on the correlation of virulence with fitness, which has mixed reports in plant pathogens (Sacristán and García‐Arenal 2008).

**FIGURE 6:**
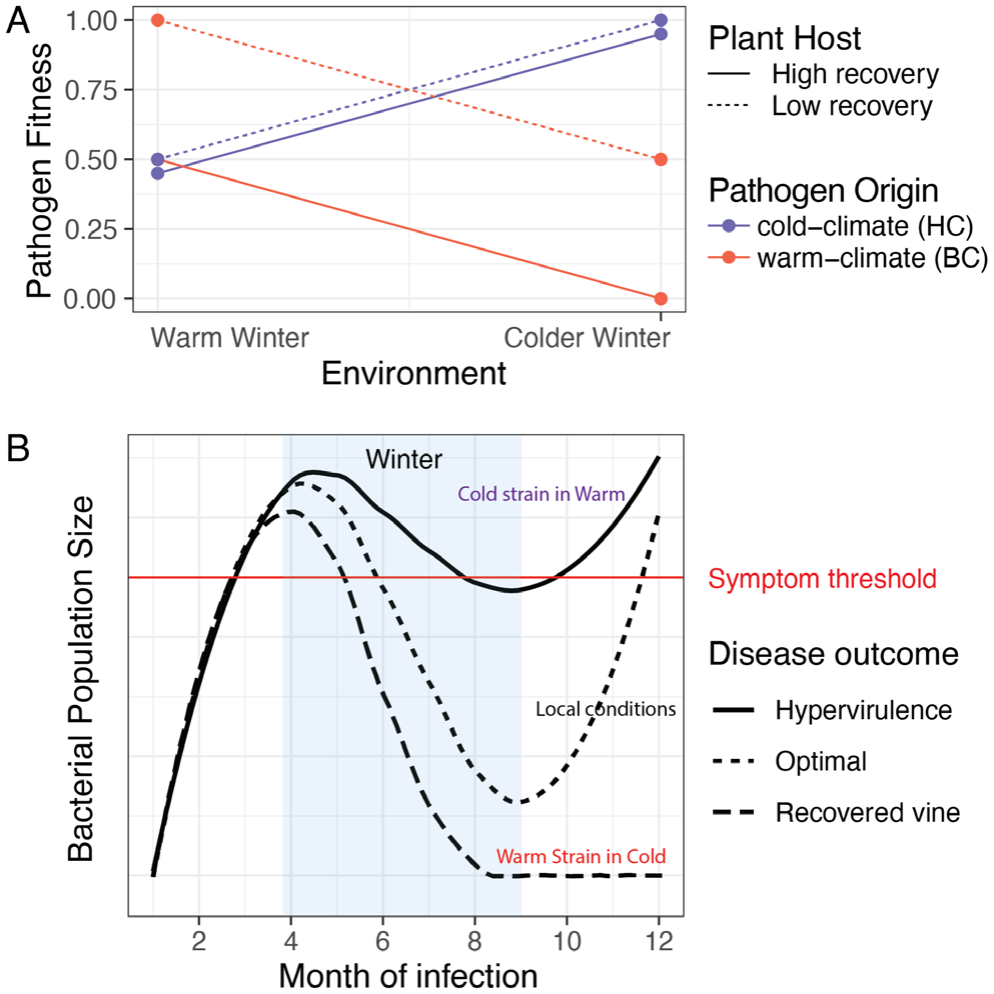
Conceptual model of pathogen fitness (A) A model of pathogen fitness across a thermal gradient, split by strain climate origin (HC: Hopland Cluster, BC: Bakersfield Cluster) and host genotype. (B) A diagram of bacterial population sizes over a 1 year infection cycle. The blue box denotes winter months.

We propose that virulence‐transmission trade‐offs limit the fitness of cold‐climate *Xf* strains in warmer regions, resulting in local adaptation. Hypervirulence (i.e., vine mortality, stunting, more severe symptoms) of cold‐climate strains after the winter is likely detrimental for pathogen fitness, as insect vectors of *Xf* discriminate against symptomatic plants (Daugherty et al. 2011, 2017). Pathogen spread is likely decreased by vector avoidance of vines with disease symptoms. Thus, in a conceptual model, the fitness of cold‐climate (HC) strains is likely decreased in warmer regions (Figure 6A). On the flip side, winter survival is generally positive for plant pathogen fitness, especially in cold climates (Figure 6A). Upon transplantation, the fitness of warm‐climate (BC) strains in a colder climate is decreased by low winter survival. This delicate balance of winter survival and virulence depends on the extent of winter population decline. The optimal strategy may be one of moderate winter decline: thus, avoiding population extinction while also avoiding the retention of large populations that result in severe symptoms or vine mortality (Figure 6B). In this conceptual model, all strains are locally adapted (Figure 6).

Our results support a link between virulence‐transmission trade‐offs and local thermal adaptation. In warm climates, a genotype that self‐restricts virulence paired with an environment that encourages symptoms results in intermediate (optimal) disease outcomes; in cold climates, a genotype with high virulence but an environment that limits symptoms also results in intermediate disease. This countergradient pattern leads to similar levels of virulence and high fitness for each pathogen population in their local conditions (Figure 6). There is some support for this enhanced growth and virulence in cold‐climate populations of fungal and oomycete plant pathogens (Chen et al. 2017; Yang et al. 2016). Virulence‐transmission trade‐offs may be particularly relevant in insect‐borne pathogens where vectors avoid symptomatic hosts. Thus, incorporating local thermal adaptation is critical to understanding the evolution of virulence in insect‐transmitted diseases and requires future empirical and theoretical research.

Host resistance or susceptibility can interact with pathogen thermal adaptation (Chen et al. 2017) and virulence evolution (Laine and Barrès 2013). Both components of fitness–winter survival and virulence–of *Xf* populations across landscapes were host‐mediated and differed by grapevine variety. Before the winter, Ciliegiolo was more susceptible to PD, based on symptom severity and development. However, higher susceptibility did not lead to low vine recovery in both strain clusters. In our second experiment, almost all Ciliegiolo plants recovered from BC infections, while no plants recovered from HC infections, and recovery was unaffected by winter temperature. On the other hand, in Cabernet Sauvignon recovery from BC strains was temperature‐dependent, with higher overwinter recovery rates in a colder climate. Recovery from HC infections was universally low, so the fitness of cold‐climate strains may be less impacted by host genotype (Figure 6A). While host effects on overwinter recovery have been demonstrated (Kahn et al. 2023; Lieth et al. 2011; Rashed et al. 2013), in warmer climates very low vine recovery from PD is expected (Burbank et al. 2023). Our results demonstrate that high recovery can occur, even in warmer climates, depending on complex interactions between environment, host genotype and pathogen genotype. This has important implications for disease management and grapevine breeding.

Here, our reciprocal transplant design isolated winter temperatures, but summer temperatures may also influence the evolution of *Xf* strains across the landscape. We observed countergradient variation in growth rate in vitro. Strains from a colder climate grew slower than warm‐climate strains at a lower temperature (20°C), but similarly at a warmer temperature (28°C). This suggests that despite evolving in a colder climate, cold‐climate strains have a higher thermal optimum; that is, they may grow faster at higher temperatures. While daytime temperatures are high in both locations, night temperatures in Bakersfield—but not Hopland—are high enough to permit rapid *Xf* growth (Figure S1). The evolution of rapid growth during the summer is a way for species to compensate for short growing seasons (Conover and Present 1990), so rapid growth at both day and night may be advantageous for *Xf* populations in Hopland. Conversely, a lower thermal optimum in warm‐climate (BC) strains may prevent uncontrolled growth (and subsequent hypervirulence) in such warm climates. After a warm fall in Bakersfield (Exp. 1), many vines infected with HC strains—but not BC strains—were dead the following year, possibly due to excessive pathogen population growth. Plant pathogen populations from Northern latitudes may evolve a higher thermal optimum to grow quickly during short windows of warm temperatures, in order to achieve large enough populations to survive colder winters.

Patterns of pathogen thermal adaptation have wide‐reaching applications for managing and predicting the spread of bacterial plant diseases. With the globalization of the plant trade, plant pathogens are increasingly moved around the globe (Santini et al. 2018). There have already been multiple recent introductions of *Xf* to Europe (Garcia et al. 2025; Gomila et al. 2019), which has led to substantial economic losses in several crop species (Schneider et al. 2020). Understanding the risk establishment of a pathogen after an introduction event is critical. In the *Xf*‐grapevine pathosystem, there seems to be minimal risk of warm‐climate strains being introduced to higher latitudes. However, an introduction of cold‐climate strains to warm areas may be devastating in the short term in the absence of further pathogen evolution–depending on virulence‐transmission trade‐offs. A deeper understanding of how local thermal adaptation intersects with virulence trade‐offs is critical to plant disease management and future risk mitigation.

## Funding

This work was supported by the National Institute of Food and Agriculture (2023‐67011‐40332) and the California Department of Food and Agriculture.

## Conflicts of Interest

The authors declare no conflicts of interest.

## Supporting information


**Data S1:** Supporting Information.

**Table S1:** Metadata on strain geographic, origin, NCBI accession, and original citation for the 10 *X. fastidiosa* strains used in this study.
**Table S2:** Statistical tables from linear mixed models built from multiple in vitro phenotype assays, including growth curves, biofilm formation, settling rate, and twitching motility.
**Table S3:** Statistical tables from various model types built from reciprocal transplant field experiments, both before and after winter exposure.
**Figure S1:** Temperature comparison at Bakersfield & Hopland: Datasets were downloaded from state‐run temperature loggers close to field sites (Hopland: Sanel Valley, Bakersfield: Arvin‐Edison). The top panel shows minimum daily air temperature in each site over 2020–2025; there is one dot for each day. The bottom panel is from hourly temperatures from 2024 in both sites, recorded at the state‐run temperature loggers. Values shown are mean, minimum and maximum for that hour of the day from all days of the month; panels are faceted by month (1–12).
**Figure S2:** Growth Curves: Growth over time was significantly greater for BC (“Bakersfield cluster”) strains than in HC (“Hopland cluster”) strains at 20°C, but not at 28°C. Each dot is the average of all replicates for 1 strain (*n* = 5 strains per cluster). Graphs are faceted by temperature (20°C or 28°C) and experimental replicate (1 through 4). The average OD (optimal density) is a metric of bacterial growth over each day of the experiment.
**Figure S3:** Endpoint Measurement: Biofilm and planktonic growth were significantly greater for BC (“Bakersfield cluster”) strains than in HC (“Hopland cluster”) strains at 20°C, but not at 28°C. Average OD (optimal density) measurements are split by experiment, type of measurement (biofilm or planktonic growth) and temperature of the experiment (20°C or 28°C). Boxplots depict the data averages from the five strains in each strain cluster. Outliers were removed to simplify the *y*‐axis scale for graphing purposes.
**Figure S4:** Settling Rate: Settling rate is a proxy for cell‐to‐cell aggregation, an important survival factor in bacterial plant pathogens. Setting rate (ΔOD) over 2 h was significantly greater in HC (“Hopland cluster”) strains than in BC (“Bakersfield cluster”) strains at both temperatures and growth media. Measurements are split by experiment, type of growth medium (PD3 or PW) and temperature of the experiment (20°C or 28°C).
**Figure S5:** Twitching Motility: Fringe width of colonies in microns (μm) on modified PW plates was measured every other day for 10 days at both 20°C and 28°C for all 10 strains. Measurements are graphed by experiment, type of measurement (biofilm or planktonic growth) and temperature of the experiment (20°C or 28°C). The number of days (*x*‐axis) reflects how long colonies were grown on plates. Outliers were removed for simplicity of graphing. All strains showed twitching motility, except for HC strain D06.
**Figure S6:** Inoculation Success: Inoculation success varied significantly by date of inoculation (*p* < 0.0001), but not by strain cluster (“HC”: Hopland Cluster, “BC”: Bakersfield Cluster). The number of vines with a successful inoculation (black: positive for Xf) out of total vines inoculated. The plot is faceted by date of inoculation (3 dates in 2023, 1 in 2024).
**Figure S7:** Populations before the winter by strain: Bacterial populations before the winter did not differ significantly by cluster (“BC”: Bakersfield Cluster, “HC”: Hopland Cluster), or by grapevine variety (“Cab”: Cabernet Sauvignon, “CE”: Ciliegiolo).
**Figure S8:** Winter temperatures at field sites: Temperature was recorded hourly in both sites with loggers. Temperatures in the top panels show a smoothed (LOESS) regression model of hourly temperature data. The bottom tables show cumulative freezing hours (< 0°C), chill hours (< 7.22°C), and fall warm hours (> 20°C) (fall: before Dec. 31st) in Exp. 1 (left: 2023–2024) and in Exp. 2 (right: 2024–2025).
**Figure S9:** Recovery Rates by Strain: Vines that recovered from *Xylella fastidiosa* infections (gray) out of the total vines that were infected (i.e., positive, “pos.”) prior to winter exposure in the previous fall (gray and red total). The top panels split by overwinter location (Bakersfield or Hopland), and the side panels split data by experimental replicate and grapevine variety (“Cab.”: Cabernet Sauvignon or “CE”: Ciliegiolo). D01, D03, D04, D06, and D07 are HC (“Hopland cluster”) strains, whereas Je115, Je60, Je66, Je77, and Je82 are BC (“Bakersfield cluster”) strains.
**Figure S10:** Populations after the winter: Experiments 1 and 2 were run on different qPCR machines, so values were not compared across experiments. Populations are from vines tested after exposure to winter in both locations. Graphs are split by experimental replicate and grapevine variety (“Cab”: Cabernet Sauvignon, “CE”: Ciliegiolo).
**Figure S11:** Scorch over time after winter: Scorch development is graphed as the decline in asymptomatic vines (“No scorch” on y‐axis) as a function of time (days in the greenhouse after winter exposure). Each graph is a unique experimental replicate/grapevine variety combination (“Cab”: Cabernet Sauvignon, “CE”: Ciliegiolo). The line type represents vines that overwintered in Bakersfield (solid) or Hopland (dashed), split by strain clusters (Red: BC Bakersfield cluster strains, Purple: HC Hopland cluster strains).

## Data Availability

Genomes and metadata for the 10 *X. fastidiosa* strains used here are publicly available on NCBI; NCBI identifiers are listed in Table S1. Raw data and analysis code for all data analyses are publicly available on GitHub.
