# Supplementary material for: Local Thermal Adaptation in an Insect‐Transmitted Plant Pathogen: The Role of Virulence Trade‐Offs
Source: Evol Appl. 2026 Jul 15;19(7):e70303. doi: 10.1111/eva.70303 (PMC13373533; doi:10.1111/eva.70303)

### Supplementary Figures

S1 - Temperature comparison at Bakersfield & Hopland

S2 - Growth curves *in vitro*

S3 - Biofilm and planktonic endpoint measurements *in vitro*

S4 - Setting rate *in vitro*

S5 - Twitching motility *in vitro*

S6 - Inoculation success

S7 - Populations before the winter by strain

S8 - Winter temperatures at field sites

S9 - Rates of recovery by strain

S10 - Populations after the winter by strain

S11 - Scorch development over time after the winter

###

### Figure S1 Temperature comparison at Bakersfield & Hopland

Datasets were downloaded from state-run temperature loggers close to field sites (Hopland: Sanel Valley, Bakersfield: Arvin-Edison). The top panel shows minimum daily air temperature in each site over 2020-2025; there is one dot for each day. The bottom panel is from hourly temperatures from 2024 in both sites, recorded at the state-run temperature loggers. Values shown are mean, minimum and maximum for that hour of the day from all days of the month; panels are faceted by month (1-12).


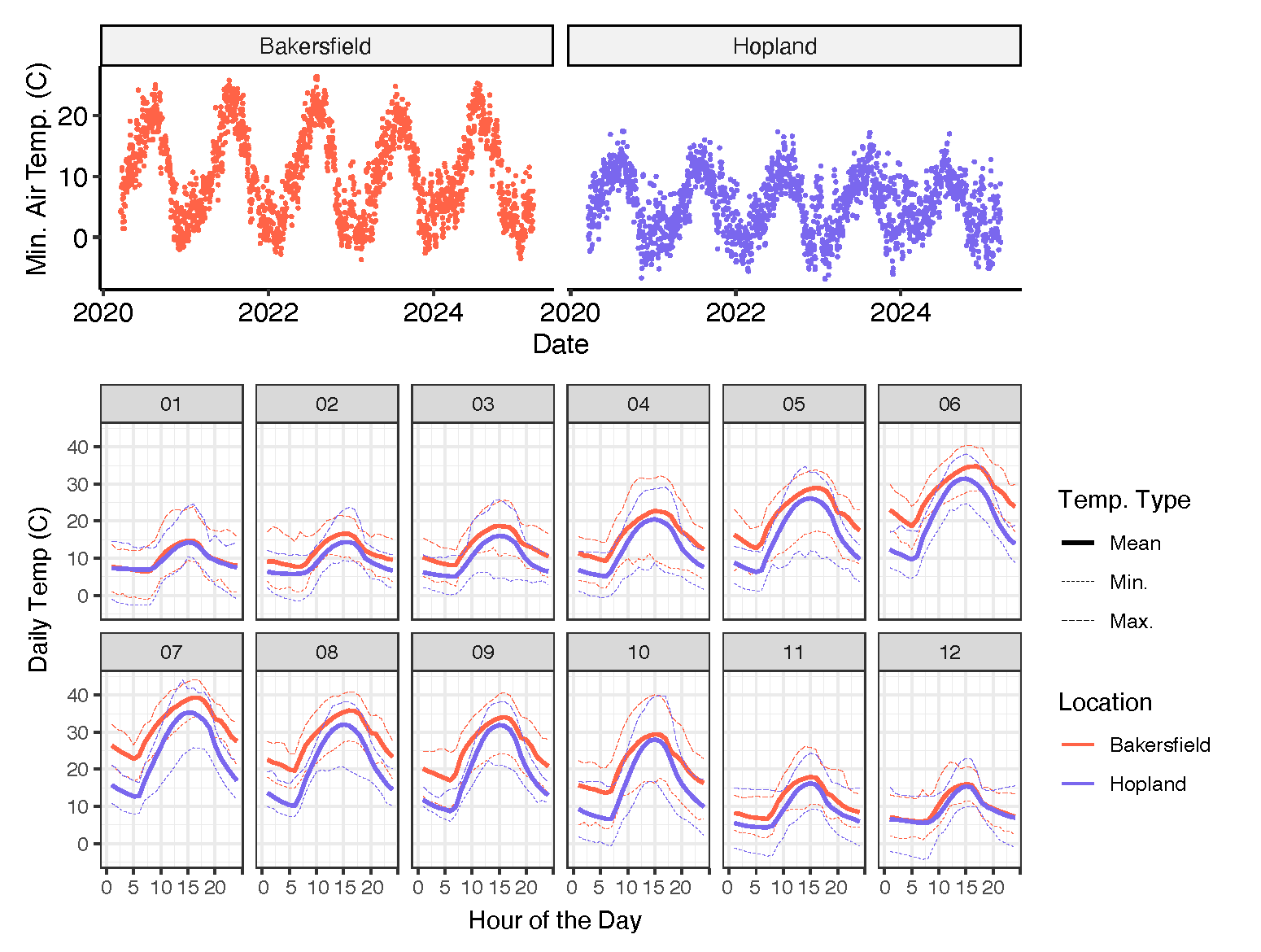


###

### Figure S2 Growth Curves

Growth over time was significantly greater for BC (“Bakersfield cluster”) strains than in HC (“Hopland cluster”) strains at 20°C, but not at 28°C. Each dot is the average of all replicates for 1 strain (n=5 strains per cluster). Graphs are faceted by temperature (20 or 28°C) and experimental replicate (1 through 4). The average OD (optimal density) is a metric of bacterial growth over each day of the experiment.


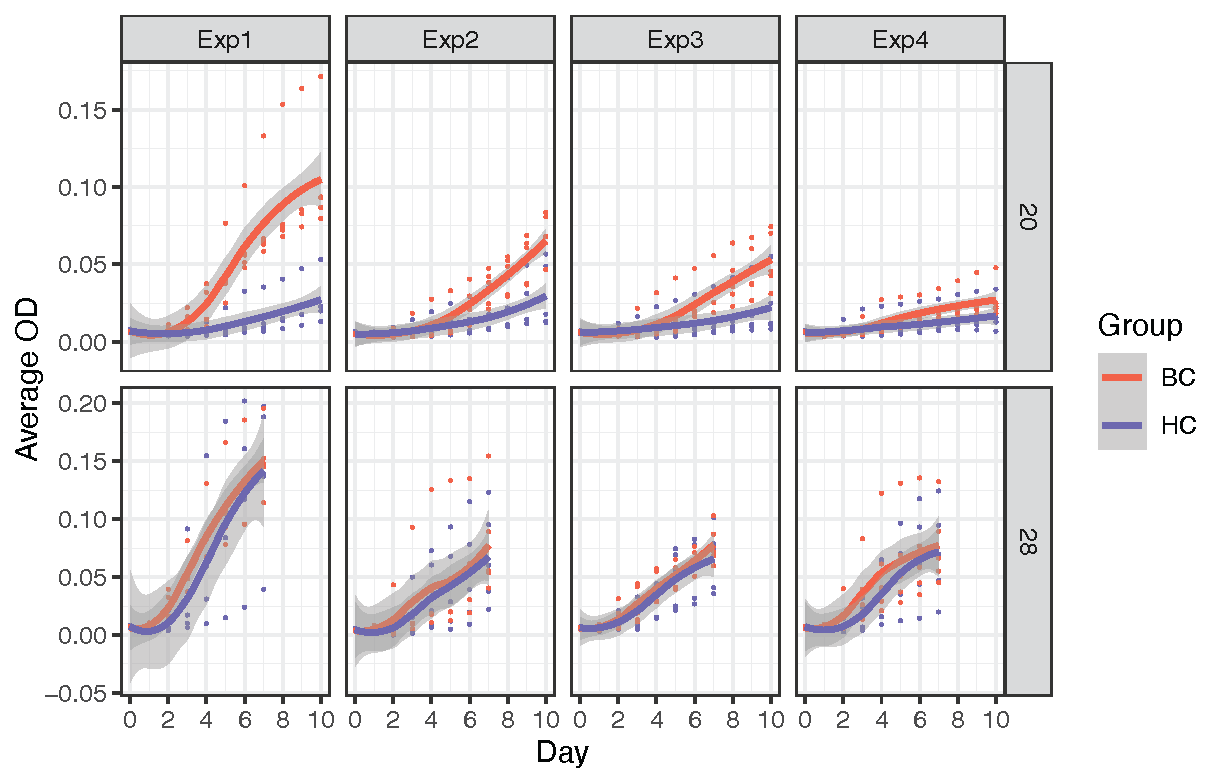


###

### Figure S3 Endpoint Measurement

Biofilm and planktonic growth were significantly greater for BC (“Bakersfield cluster”) strains than in HC (“Hopland cluster”) strains at 20°C, but not at 28°C. Average OD (optimal density) measurements are split by experiment, type of measurement (biofilm or planktonic growth) and temperature of the experiment (20°C or 28°C). Boxplots depict the data averages from the five strains in each strain cluster. Outliers were removed to simplify the y-axis scale for graphing purposes.


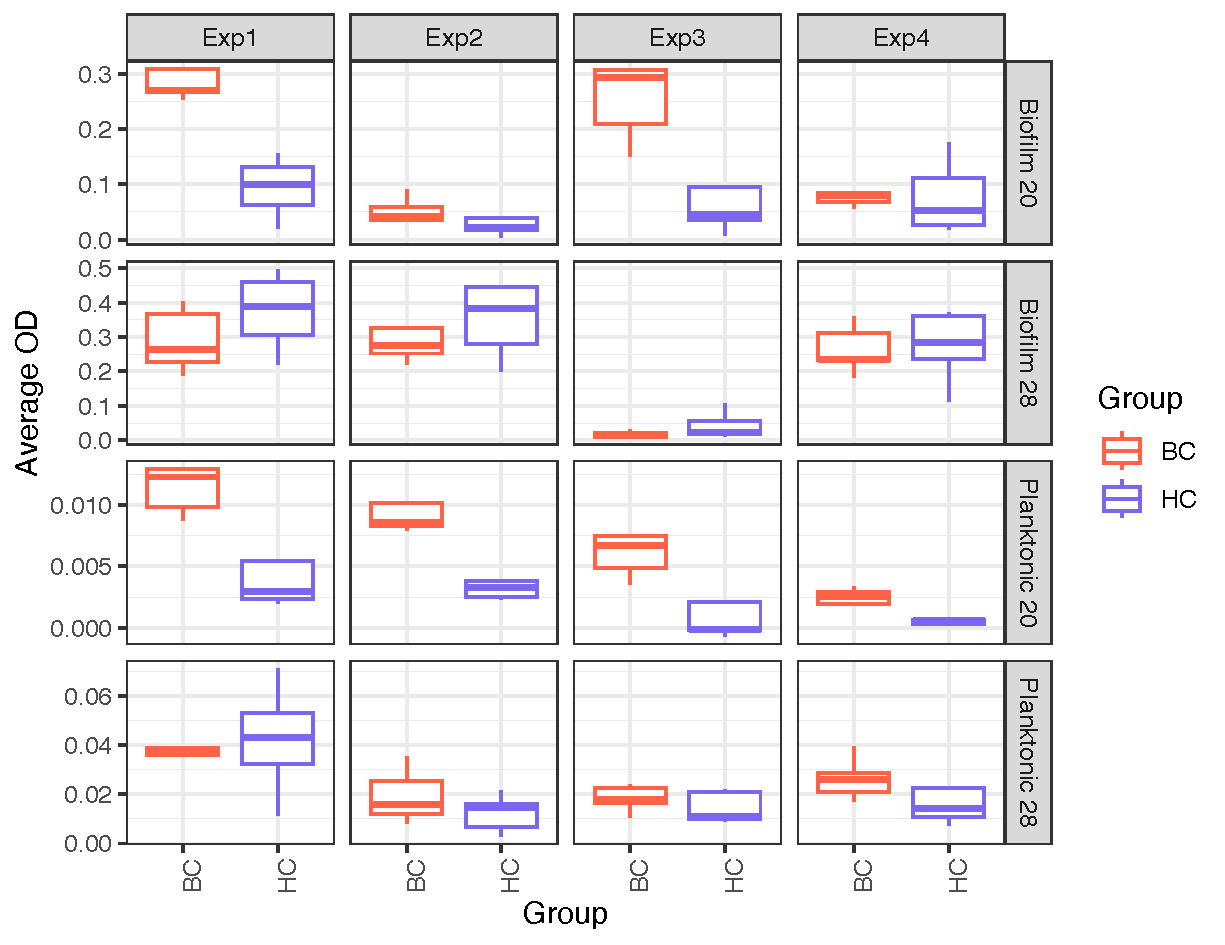


###

### Figure S4 Settling Rate

Settling rate is a proxy for cell-to-cell aggregation, an important survival factor in bacterial plant pathogens. Setting rate (ΔOD) over two hours was significantly greater in HC (“Hopland cluster”) strains than in BC (“Bakersfield cluster”) strains at both temperatures and growth media. Measurements are split by experiment, type of growth medium (PD3 or PW) and temperature of the experiment (20°C or 28°C).


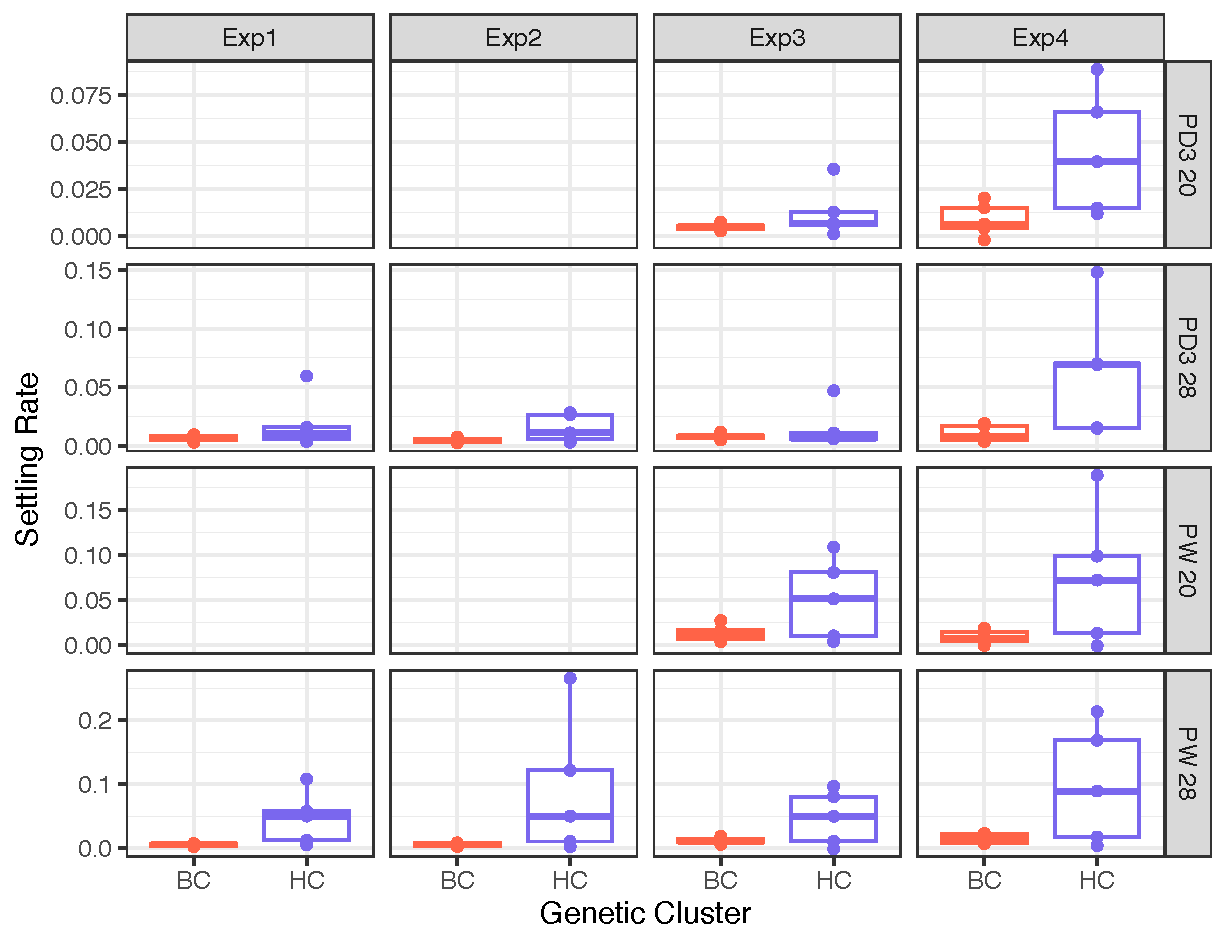


###

### Figure S5 Twitching Motility

Fringe width of colonies in microns (µm) on modified PW plates was measured every other day for 10 days at both 20°C and 28°C for all ten strains. Measurements are graphed by experiment, type of measurement (biofilm or planktonic growth) and temperature of the experiment (20 or 28°C). The number of days (x-axis) reflects how long colonies were grown on plates. Outliers were removed for simplicity of graphing. All strains showed twitching motility, except for HC strain D06.


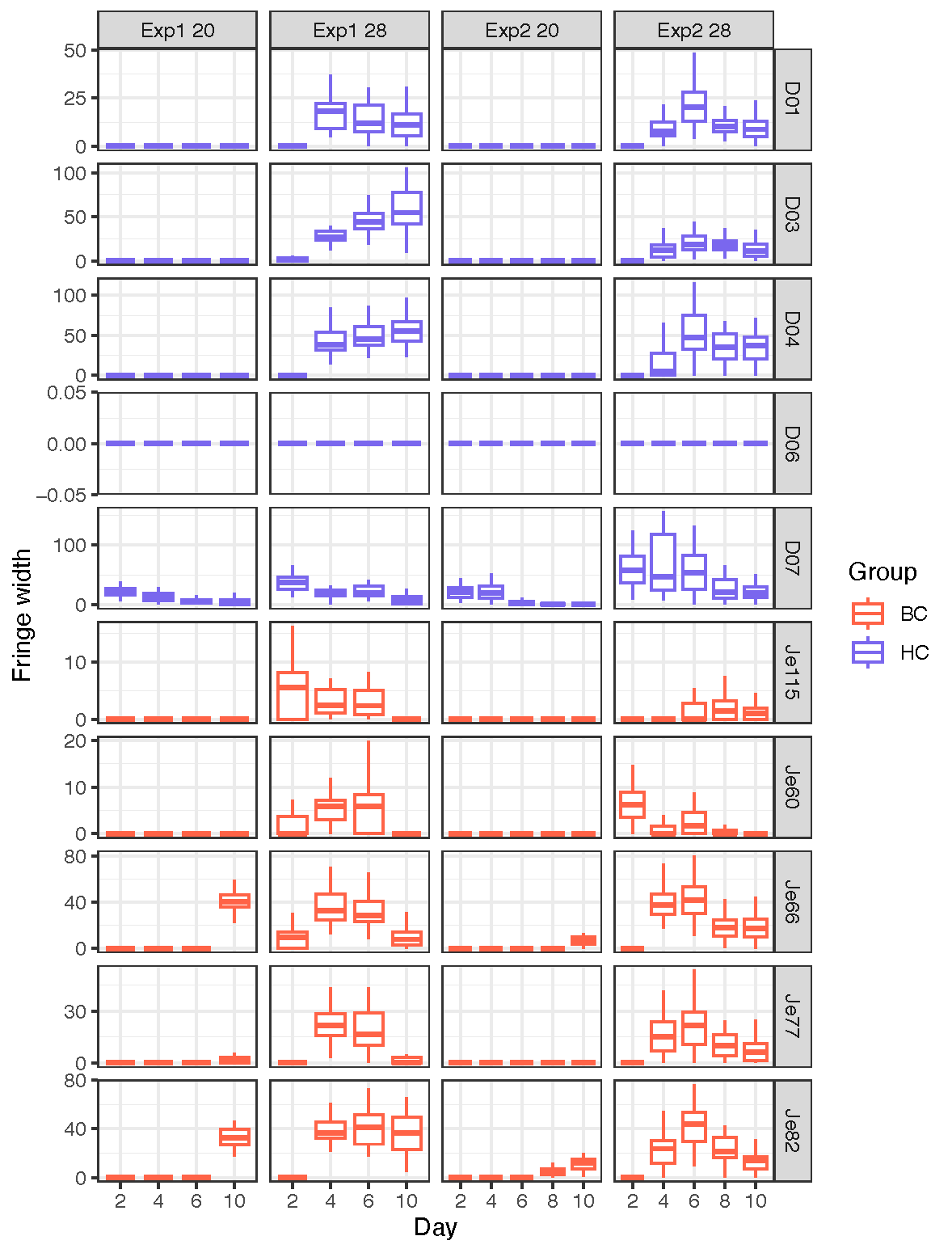


### Figure S6 Inoculation Success

Inoculation success varied significantly by date of inoculation (p<0.0001), but not by strain cluster (“HC”: Hopland Cluster, “BC”: Bakersfield Cluster). The number of vines with a successful inoculation (black: positive for *Xf*) out of total vines inoculated. The plot is faceted by date of inoculation (3 dates in 2023, 1 in 2024).


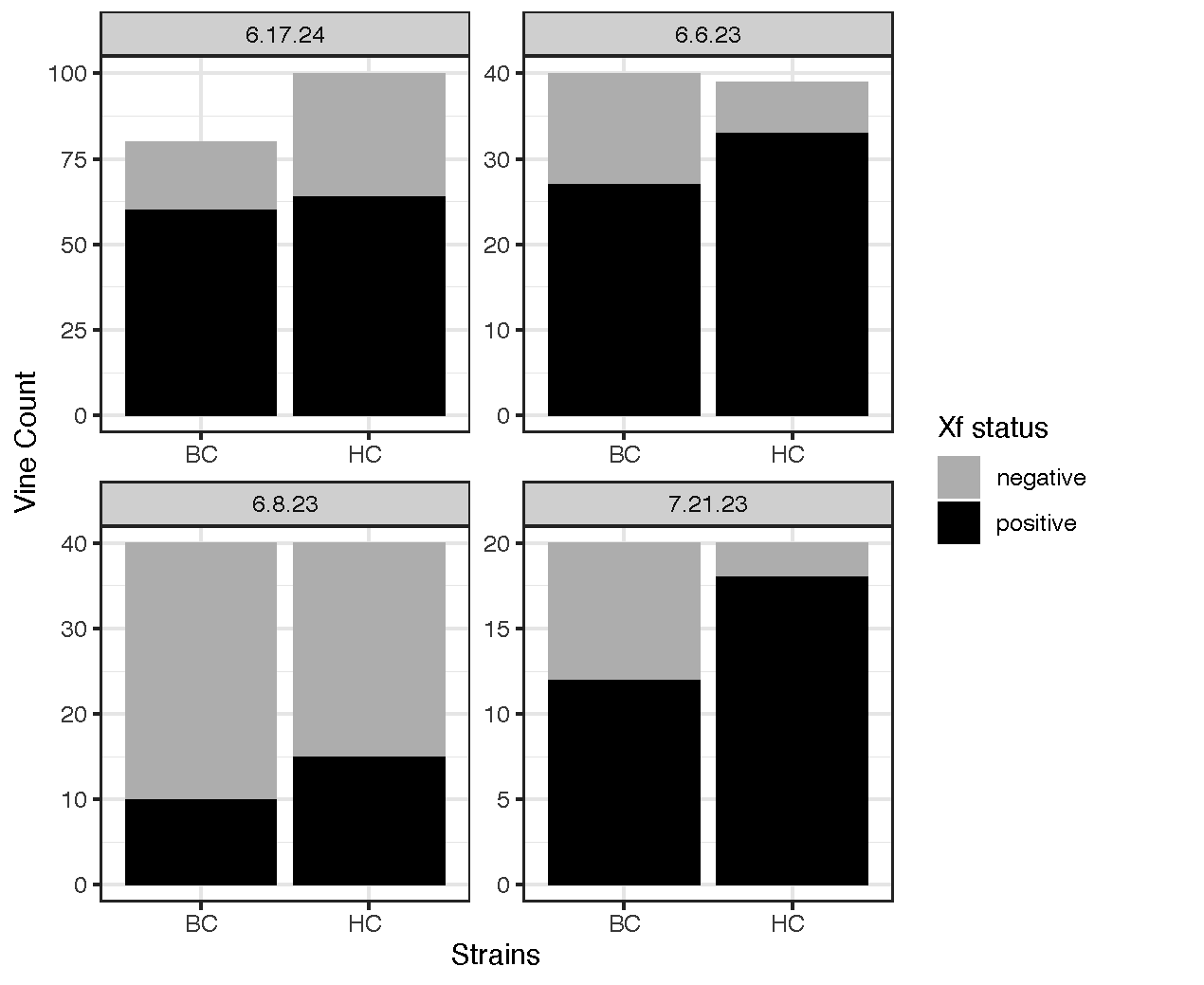


###

###

### Figure S7 Populations before the winter by strain

Bacterial populations before the winter did not differ significantly by cluster (“BC”: Bakersfield Cluster, “HC”: Hopland Cluster), or by grapevine variety (“Cab”: Cabernet Sauvignon, “CE”: Ciliegiolo).


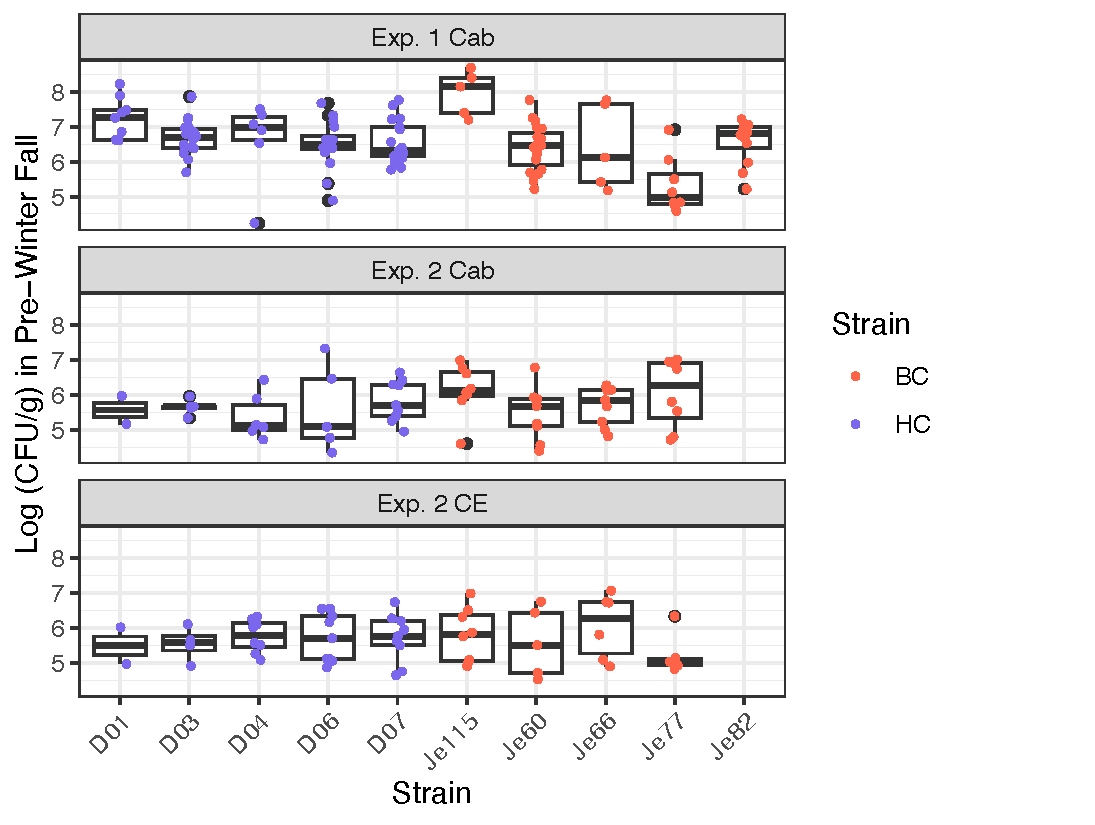


###

### Figure S8 Winter temperatures at field sites

Temperature was recorded hourly in both sites with loggers. Temperatures in the top panels show a smoothed (LOESS) regression model of hourly temperature data. The bottom tables show cumulative freezing hours (<0°C), chill hours (<7.22°C), and fall warm hours (>20°C) (fall: before Dec. 31st) in Exp. 1 (left: 2023-2024) and in Exp. 2 (right: 2024-2025).

###
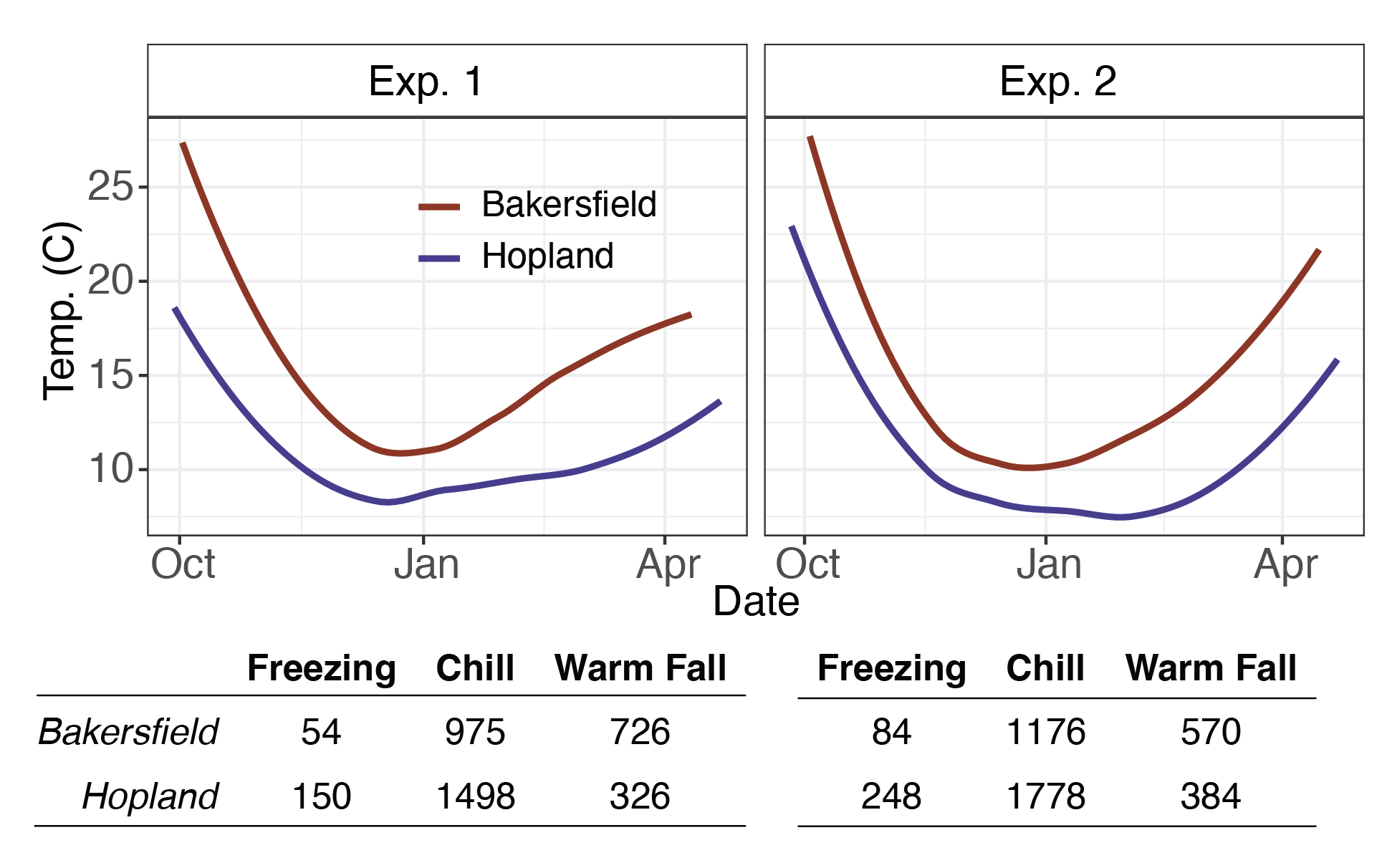


###

### Figure S9 Recovery Rates by Strain

Vines that recovered from *Xylella fastidiosa* infections (grey) out of the total vines that were infected (*i.e.* positive, “pos.”) prior to winter exposure in the previous fall (grey and red total). The top panels split by overwinter location (Bakersfield or Hopland), and the side panels split data by experimental replicate and grapevine variety (“Cab.”: Cabernet Sauvignon or “CE”: Ciliegiolo). D01, D03, D04, D06, and D07 are HC (“Hopland cluster”) strains, whereas Je115, Je60, Je66, Je77, and Je82 are BC (“Bakersfield cluster”) strains.


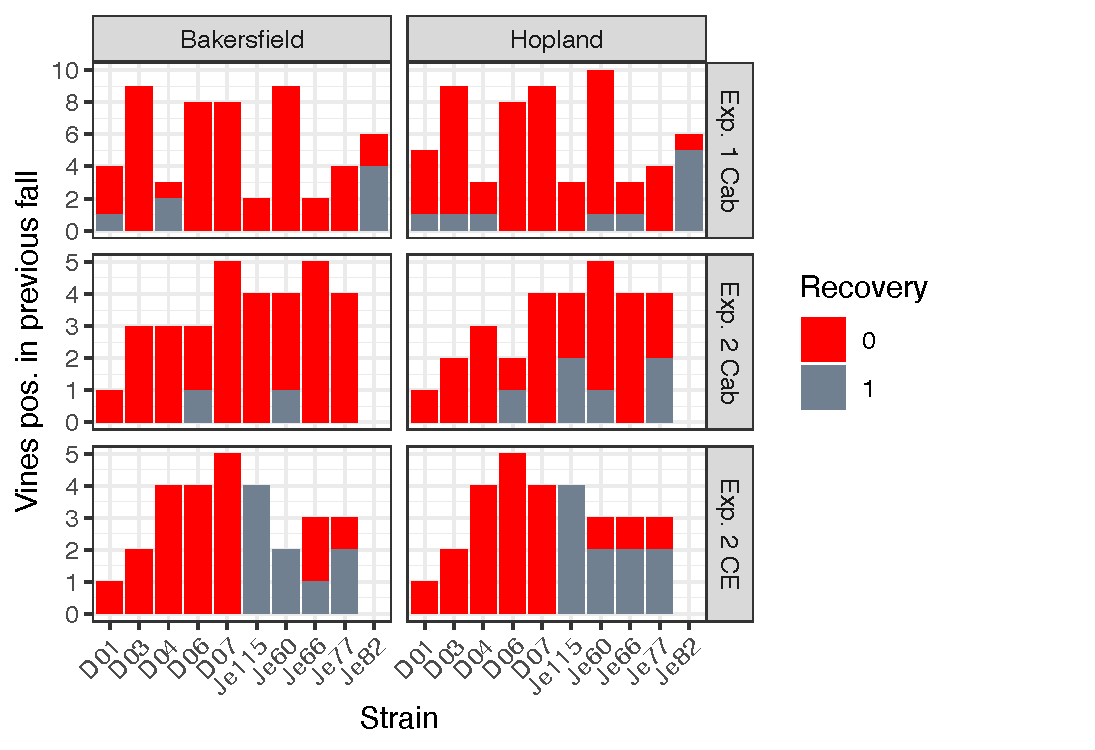


###

###

### Figure S10 Populations after the winter

Experiments 1 and 2 were run on different qPCR machines, so values were not compared across experiments. Populations are from vines tested after exposure to winter in both locations. Graphs are split by experimental replicate and grapevine variety (“Cab”: Cabernet Sauvignon, “CE”: Ciliegiolo).


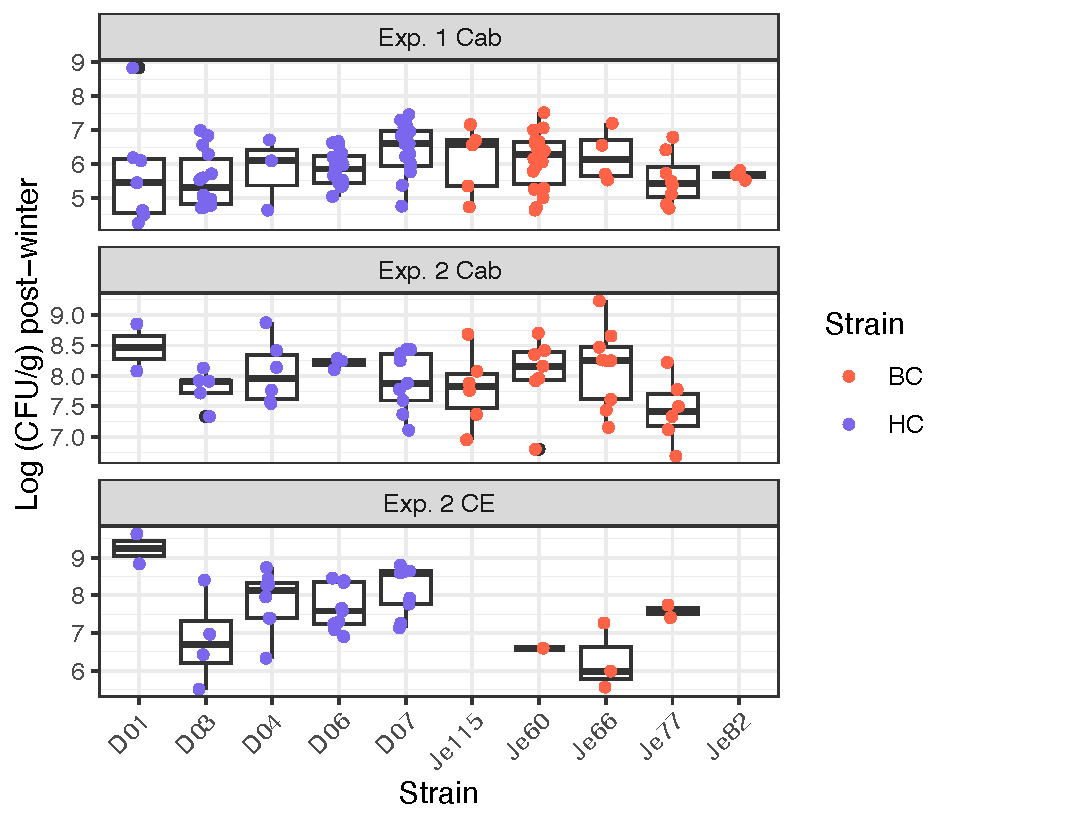


###

### Figure S11 Scorch over time after winter

Scorch development is graphed as the decline in asymptomatic vines (“No scorch” on y-axis) as a function of time (days in the greenhouse after winter exposure). Each graph is a unique experimental replicate / grapevine variety combination (“Cab”: Cabernet Sauvignon, “CE”: Ciliegiolo). The line type represents vines that overwintered in Bakersfield (solid) or Hopland (dashed), split by strain clusters (Red: BC Bakersfield cluster strains, Purple: HC Hopland cluster strains).


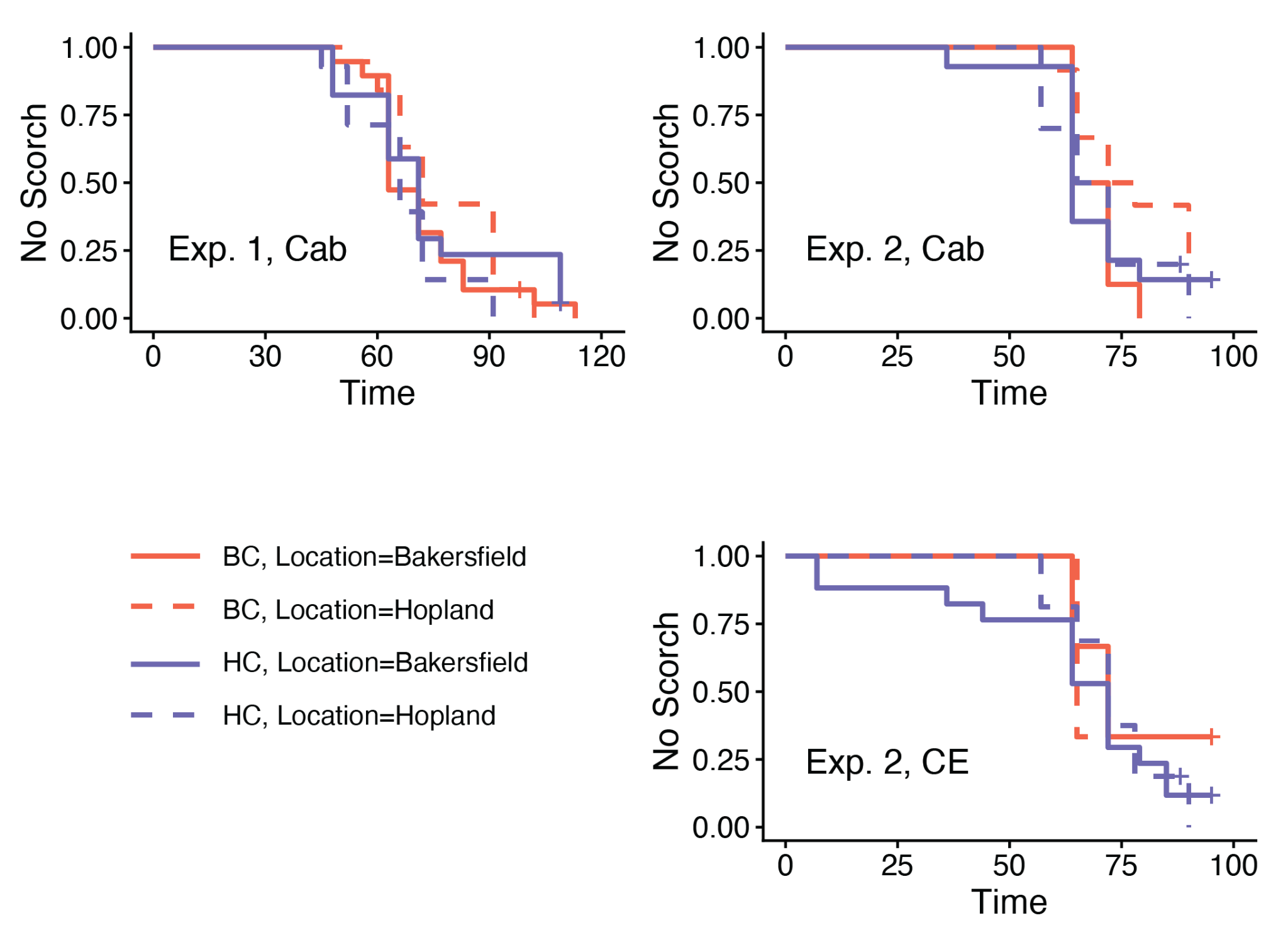

Supplement: Supplementary file 2 — Table S1: Metadata on strain geographic, origin, NCBI accession, and original citation for the 10 X. fastidiosa strains used in this study. Table S2: Statistical tables from linear mixed models built from multiple in vitro phenotype assays, including growth curves, biofilm formation, settling rate, and twitching motility. Table S3: Statistical tables from various model types built from reciprocal transplant field experiments, both before and after winter exposure. Figure S1: Temperature comparison at Bakersfield & Hopland: Datasets were downloaded from state‐run temperature loggers close to field sites (Hopland: Sanel Valley, Bakersfield: Arvin‐Edison). The top panel shows minimum daily air temperature in each site over 2020–2025; there is one dot for each day. The bottom panel is from hourly temperatures from 2024 in both sites, recorded at the state‐run temperature loggers. Values shown are mean, minimum and maximum for that hour of the day from all days of the month; panels are faceted by month (1–12). Figure S2: Growth Curves: Growth over time was significantly greater for BC (“Bakersfield cluster”) strains than in HC (“Hopland cluster”) strains at 20°C, but not at 28°C. Each dot is the average of all replicates for 1 strain (n = 5 strains per cluster). Graphs are faceted by temperature (20°C or 28°C) and experimental replicate (1 through 4). The average OD (optimal density) is a metric of bacterial growth over each day of the experiment. Figure S3: Endpoint Measurement: Biofilm and planktonic growth were significantly greater for BC (“Bakersfield cluster”) strains than in HC (“Hopland cluster”) strains at 20°C, but not at 28°C. Average OD (optimal density) measurements are split by experiment, type of measurement (biofilm or planktonic growth) and temperature of the experiment (20°C or 28°C). Boxplots depict the data averages from the five strains in each strain cluster. Outliers were removed to simplify the y‐axis scale for graphing purposes. Figure S4: Settli [file EVA-19-e70303-s001.docx]
